# Temporal and spatial regulation of protein cross-linking by the pre-assembled substrates of a *Bacillus subtilis* spore coat transglutaminase

**DOI:** 10.1371/journal.pgen.1007912

**Published:** 2019-04-08

**Authors:** Catarina G. Fernandes, Diogo Martins, Guillem Hernandez, Ana L. Sousa, Carolina Freitas, Erin M. Tranfield, Tiago N. Cordeiro, Mónica Serrano, Charles. P. Moran, Adriano O. Henriques

**Affiliations:** 1 Microbial Development Group, Instituto de Tecnologia Química e Biológica António Xavier, Universidade Nova de Lisboa, ITQB-NOVA, Oeiras, Portugal; 2 Dynamic Structural Biology Laboratory, Instituto de Tecnologia Química e Biológica António Xavier, Universidade Nova de Lisboa, ITQB-NOVA, Oeiras, Portugal; 3 Electron Microscopy Facility, Instituto Gulbenkian de Ciência, Oeiras, Portugal; 4 Emory University School of Medicine, Atlanta GA, United States of America; Indiana University, UNITED STATES

## Abstract

In many cases protein assemblies are stabilized by covalent bonds, one example of which is the formation of intra- or intermolecular ε-(γ-glutamyl)lysil cross-links catalyzed by transglutaminases (TGases). Because of the potential for unwanted cross-linking reactions, the activities of many TGases have been shown to be tightly controlled. Bacterial endospores are highly resilient cells in part because they are surrounded by a complex protein coat. Proteins in the coat that surrounds *Bacillus subtilis* endospores are crosslinked by a TGase (Tgl). Unlike other TGases, however, Tgl is produced in an active form, and efficiently catalyzes amine incorporation and protein cross-linking in vitro with no known additional requirements. The absence of regulatory factors raises questions as to how the activity of Tgl is controlled during spore coat assembly. Here, we show that substrates assembled onto the spore coat prior to Tgl production govern the localization of Tgl to the surface of the developing spore. We also show that Tgl residues important for substrate recognition are crucial for its localization. We identified the glutamyl (Q) and lysil (K) substrate docking sites and we show that residues on the Q side of Tgl are more important for the assembly of Tgl than those on the K side. Thus, the first step in the reaction cycle, the interaction with Q-substrates and formation of an acyl-enzyme intermediate, is also the determinant step in the localization of Tgl. Consistent with the idea that Tg exerts a “spotwelding” activity, cross-linking pre-formed assemblies, we show that C30 is an oblong hexamer in solution that is cross-linked *in vitro* into high molecular weight forms. Moreover, during the reaction, Tgl becomes part of the cross-linked products. We suggest that the dependency of Tgl on its substrates is used to accurately control the time, location and extent of the enzyme´s activity, directed at the covalent fortification of pre-assembled complexes at the surface of the developing spore.

## Author summary

The orderly recruitment of proteins during the assembly of complex macromolecular structures poses challenges throughout cell biology. During endospore development in the bacterium *Bacillus subtilis* at least 80 proteins synthesized in the mother cell are assembled around the developing spore to form a protective coat. Regulation of coat gene expression has been described in detail but it is unknown how the information encoded by the structures of the proteins guide their assembly. We have examined the assembly of a transglutaminase, Tgl, which introduces ε-(γ-glutamyl)lysil cross-links in coat protein substrates. We describe with molecular detail a substrate-driven assembly model that directs the enzyme to the locations of its substrates where, as we suggest, it exerts a “spotwelding” activity to fortify pre-assembled complexes. The catalytic cysteine, located in a tunnel that spans the Tgl structure, first forms an acyl enzyme intermediate with a glutamine (Q) donor substrate. Then, it engages a lysine (K) donor substrate to form the cross-linked product. We have identified the Q and K acceptor ends of the Tgl tunnel, and we show that substitutions in substrate recognition residues at the Q side impair assembly more strongly than at the K side. Thus, assembly of Tgl parallels its catalytic cycle, directing the enzyme to the pre-formed complexes that are to be cross-linked.

## Introduction

Protein function is often restricted to specific cellular locations, in both eukaryotes or prokaryotes, and knowledge of the pathways governing protein localization is essential to understand protein function (reviewed by [1]). The formation of supramolecular protein assemblies, for instance, requires targeting pathways that direct the various components to the appropriate cellular location or locations at the right time [1]. In many cases, protein assemblies, whether static or dynamic, are formed through non-covalent interactions of their components. In other cases, protein assemblies are stabilized by covalent bonds, one example of which is the formation of intra- or intermolecular ε-(γ-glutamyl)lysil cross-links catalyzed by transglutaminases (TGases) [2]. TGases are involved in blood clotting processes, the organization of the extracellular matrix, tissue and bone mineralization, cell adhesion, stabilization of dermo-epidermal junctions, or the cross-linking of eye lens proteins [2–4]. Because of the potential for unwanted cross-linking reactions, the activity of the human and human-like enzymes is tightly controlled as illustrated by human factor XIIIa (FXIIIa), a TGase responsible for the cross-linking of fibrin in the last stages of the blood coagulation cascade [5]. Factor XIII (FXIII), the zymogenic inactive form of FXIIIa, exists in the plasma as an A_2_B_2_ heterotetramer, in which the A_2_ subunits correspond to the TGase zymogen, while B_2_ are non-enzymatic; most of the FXIII in the plasma is bound to fibrinogen, the fibrin precursor, by its B_2_ subunits [5]. Thus, the inactive TGase is associated with its substrate precursor during the coagulation cascade ensuring proper localization of the enzyme for the formation of the blood clot. FXIII activation will then be elicited by thrombin processing and dissociation of the B_2_ subunits. Fibrinogen is cleaved by thrombin and fibrin then self-assembles into protofibrils and fibers held together by non-covalent bonds, eventually forming a clot network. In a classical example of “spotwelding” activity, FXIIIa cross-links and thereby stabilizes the fibrin polymer, a process essential for haemostasis [2, 5]. Furthermore, the association of fibrin chains stimulates the activity of FXIIIa at specific cross-linking sites, a process that has been termed “assembly-driven regulation of cross-linking” [2, 5].

**Fig 1:**
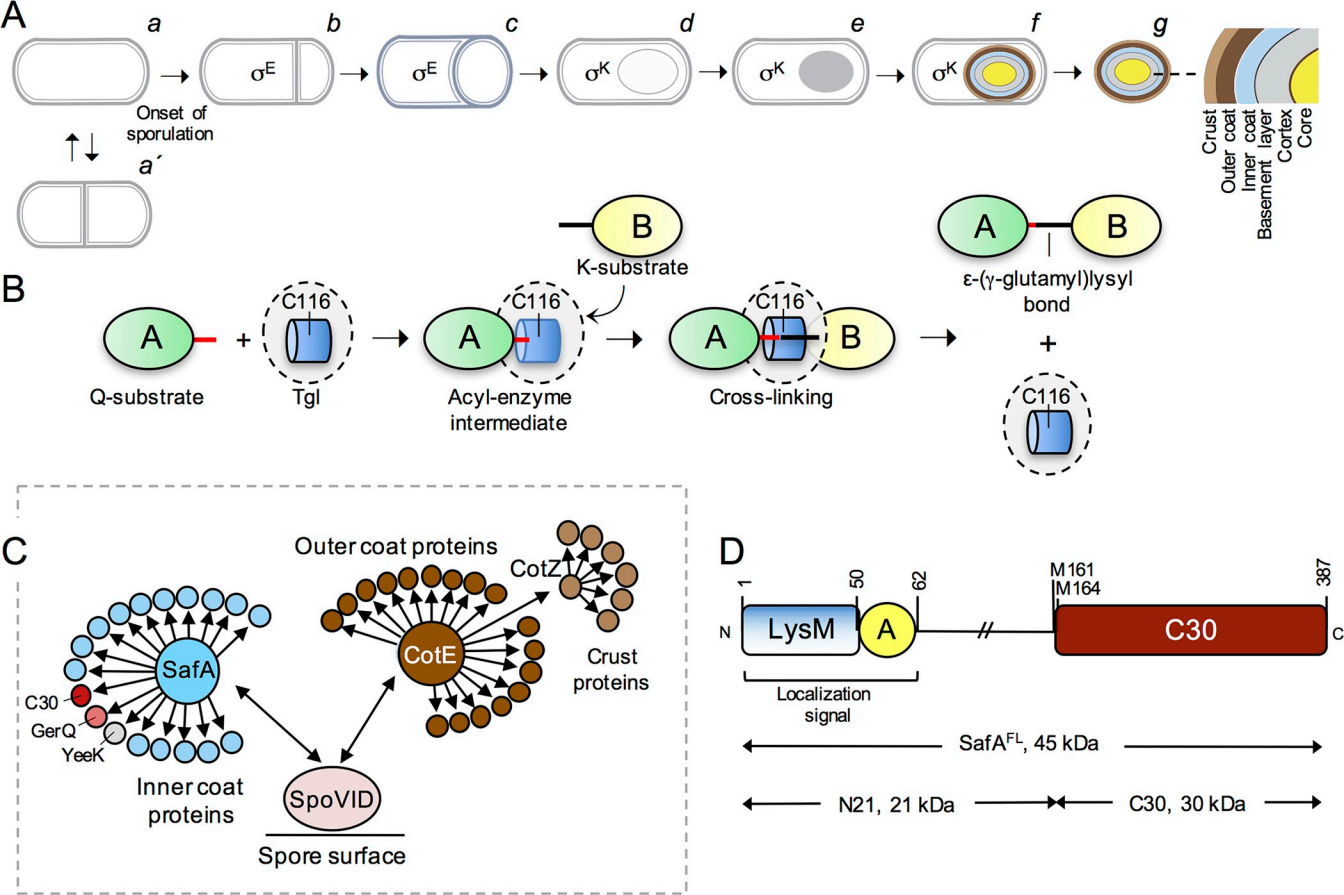
Activity and localization of Tgl during sporulation. **A**: Vegetative cells of *B*. *subtilis* grow and divide at midcell (*a* and *a*’) but at the onset of sporulation the cells switch to a polar division that forms a smaller forespore (the future spore) and a larger mother cell (*b*). Later stages in spore differentiation include; *c*, initiation of engulfment; *d*, engulfment completion; *e*, formation of phase dark spores; *f*, final stages in the assembly of the spore protective structure; *g*, free spores. The main spore structures are represented at the end of the sequence. The stages in which the mother cell-specific regulatory proteins σ^E^ and σ^K^ are active are also shown. **B**: Reaction cycle of Tgl. The active site (only the catalytic Cys116 residues is represented) is located within a tunnel that transverses the molecule from side to side. A Gln-containing substrate (the acceptor or Q-substrate in protein A) binds to the enzyme from one side of the tunnel and forms an acyl-enzyme intermediate with the catalytic Cys116. A Lys-containing substrate (the donor or K-substrate in protein B, but note that inter-molecular reactions are also possible) then approaches the enzyme from the opposite side of the tunnel and attacks the thiolester bond, leading to the formation of a ε-(γ-glutamyl)lysil isopeptide bond between A and B. Lastly, the cross-linked substrate is released [14]. **C**: The figure illustrates the role of morphogenetic proteins SafA, CotE and CotZ in recruiting the inner, outer and crust proteins during spore coat assembly; SafA and CotE interact directly with the encasement protein SpoVID. SpoVM and SpoIVA are not represented for simplicity (see text for details). C30, GerQ (σ^E^-controlled, pink) and YeeK (σ^K^-dependent, grey) are SafA-dependent, inner coat proteins. **D**: structural organization of SafA. The full-length protein, SafA^FL^, carries a localization signal at the N-terminal end, formed by a peptidoglycan-binding LysM domain and region A, which binds to the encasement protein SpoVID [28, 29, 49]. The internal Met codons M161 and M164 can direct production of a short form of the protein, termed C30, through internal translation of the *safA* mRNA. C30 binds to SafA^FL^ and is recruited to the coat in this way. Concomitant with the production of C30, a third form of the protein, N21, is also formed [28].

Species of the *Bacillus* and *Clostridium* genera and related organisms, have the ability to differentiate into dormant endospores (spores for simplicity) arguably one of the most resilient cell types found in nature [6–8] (Fig 1A). Spores are composed of three main concentric compartments: the core, which contains a copy of the genome; the cortex, composed of a modified form of peptidoglycan, and the coat, which surrounds the cortex [9–11] (Fig 1A). In the best-studied spore-forming organism, *B*. *subtilis*, the coat is assembled from over 70 polypeptides and has a role in spore protection and germination [9–13]. Perhaps not surprisingly, one of the proteins recruited to the coat layers, Tgl, is a TGase. Tgl shows the NlpC/P60 catalytic core characteristic of TGases and a dual, partially redundant catalytic dyad located in a tunnel that transverses the molecule from side to side [14]. In the reaction cycle, shared by all TGases, a glutamyl (Q or acceptor) substrate binds to the enzyme at one side of the tunnel and forms an acyl-enzyme intermediate with the catalytic Cys116 (Fig 1B). Only then, a lysil (K or donor) substrate approaches the enzyme from the opposite side of the tunnel and attacks the thiolester bond, leading to the formation of a ε-(γ-glutamyl)lysil isopeptide bond [2, 5] (Fig 1B). Importantly, unlike other TGases, Tgl is produced in an active form, and efficiently catalyzes amine incorporation and protein cross-linking in vitro with no known additional requirements [14, 15]. Tgl is a small and structurally simple TGase, and we have argued that it embodies the minimal structural requirements for protein cross-linking [14, 15]. The absence of regulatory factors raises questions as to how the activity of Tgl is controlled during spore coat assembly.

Sporulation takes place in a sporangium formed by a larger mother cell and a smaller forespore that will become the future spore (Fig 1A). Assembly of the coat is mainly a function of the mother cell and begins soon after asymmetric division with the expression of genes coding for early spore coat proteins under the control of RNA polymerase sigma subunit σ^E^ [9, 10, 16]. After asymmetric division the forespore is engulfed by the mother cell and becomes isolated from the external medium (Fig 1A). Engulfment completion coincides with the onset of transcription of the genes coding for late spore coat proteins, under the control of σ^K^ [9–11]. σ^K^ also triggers cortex polymerization; soon after, the spore development process is finalized through lysis of the mother cell and release of the spore (Fig 1A). The coat is formed by four main layers: a basement layer which sits on the cortex and is surrounded by an inner and outer coat layers and finally by the crust [9–11] (Fig 1A). Four morphogenetic proteins that localize to the forespore surface as engulfment begins, create an organizational scaffold for the construction of the different coat layers [10, 11, 16, 17]. Formation of the basement layer requires SpoIVA, tethered to the forespore membrane by a small peptide, SpoVM, which recognizes the positive curvature of the forespore outer membrane [18–21]. SafA and CotE, are necessary for inner and outer coat formation, respectively, and CotZ governs formation of the crust [22–27] (Fig 1C). These proteins are recruited by SpoIVA and bring to the spore surface the proteins that form the various coat layers. In a second step termed encasement, that requires SpoVM and another morphogenetic protein, SpoVID, the coat proteins migrate around the forespore, in successive waves determined in part by the activities of σ^E^ and σ^K^ and their auxiliary transcription factors [17]. SafA, CotE and CotZ appear to function as hubs, *i*.*e*., they may interact directly with several of the coat or crust proteins whose assembly they direct (Fig 1C). The C30 protein, for instance, interacts directly with SafA [27]. C30 is formed by internal translation initiation at Met codons 161 and 164 of the *safA* mRNA [28] (Fig 1D). Since it lacks the localization signals found at the N-terminus of the full-length protein, which include a LysM domain and a short region (region A) that both mediate an interaction with SpoVID, C30 relies on the interaction with SafA^FL^ for assembly [28–30]. SafA^FL^ and C30, as well as two other inner coat SafA-dependent proteins, YeeK and GerQ, are substrates for Tgl [31–35] (Fig 1C). SafA^FL^ and C30, as well as Tgl itself, are found mainly in the cortex and inner coat [27, 35, 36]. SafA is a key factor in recruitment of Tgl to the inner coat, but is indispensable for its association with the cortex; this pathway is auto-regulatory, in that following recruitment by SafA, Tgl then cross-links SafA^FL^ and C30 in both the coat and the cortex [35].

Here, we show how the activity of Tgl is spatially restricted to the surface of the developing spore. We show that assembly of Tgl depends on the prior assembly of its substrates. Conversely, we have uncovered residues in the vicinity of the active site of Tgl involved in enzyme-substrate interactions that are critical for the assembly of Tgl. We identify the Q and K sides of Tgl, and we show that Q-side residues make a more important contribution to assembly of the enzyme than K-side residues. Hence, the steps in Tgl assembly parallel the steps in the enzyme´s reaction cycle [35]. Supporting a model in which Tgl exerts a “spotwelding” activity to covalently fortify pre-assembled complexes, we show C30 is a hexamer that is cross-linked by Tgl into high molecular weight forms. Moreover, we show that the enzyme itself becomes part of the cross-linked products. Together, our results suggest that the dependency on the prior localization of its substrates spatially and temporally restricts the activity of Tgl, ensuring that pre-assembled complexes are cross-linked at the spore surface.

## Results

### The YeeK and GerQ substrates control spore encasement by Tgl-CFP

**Fig 2:**
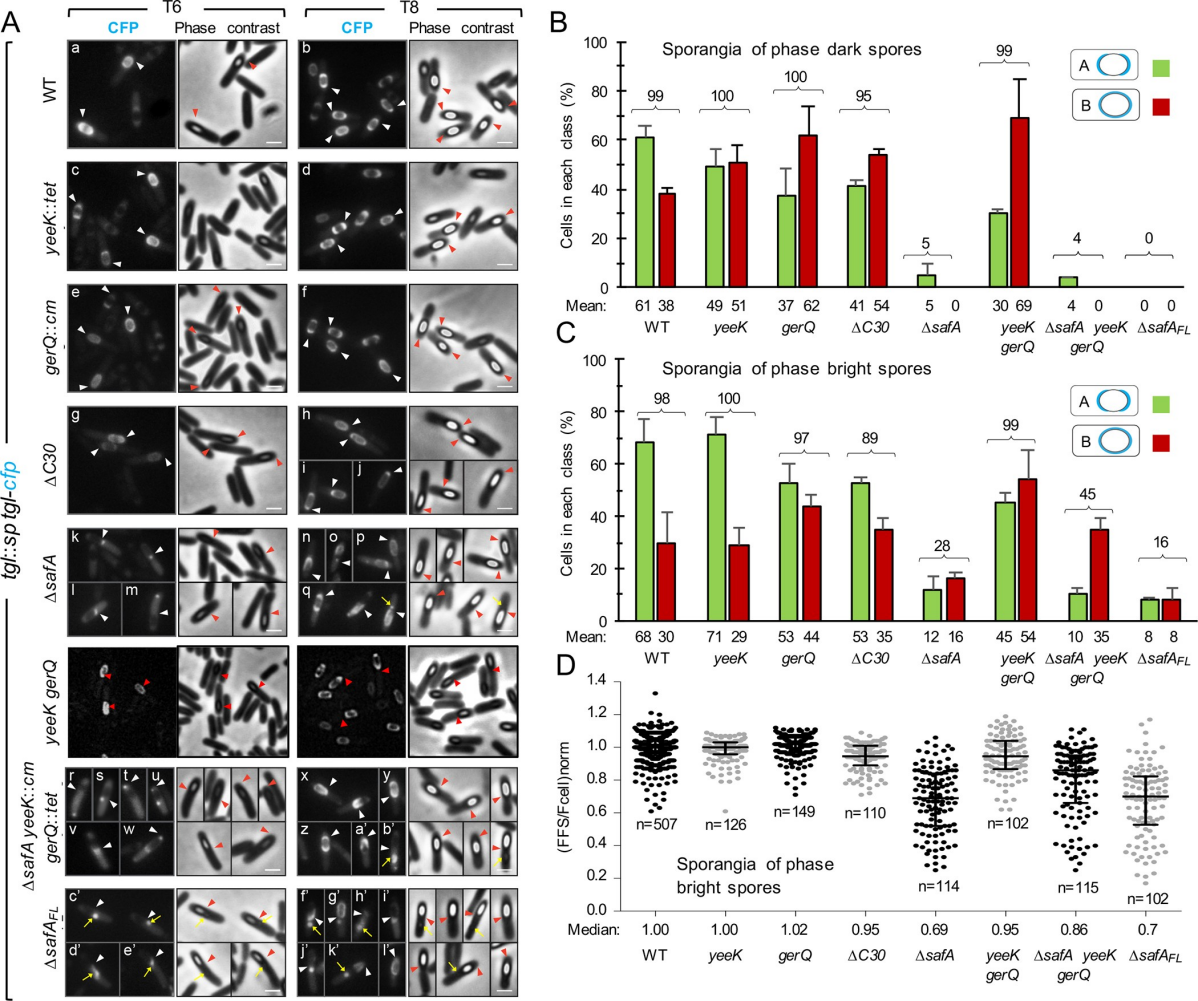
The localization of Tgl depends on its substrates. **A**: Samples of different strains expressing *tgl-cfp* were collected 6 and 8 hours after induction of sporulation by resuspension and processed for phase contrast (PC) and fluorescence microscopy. All strains bear an insertional *tgl*::*sp* allele and *tgl-cfp* at the *amyE* locus, in addition to the indicated mutations. The *tgl*::*sp* mutant producing Tgl-CFP from *amyE* serves as the reference strain and is referred to in the figure as the WT. The Δ*C30* strain bears the *safA*^M161/164A^ allele and is unable to produce C30 by internal translation; the Δ*safA*_FL_ strain bears the *safA*^F155STOP^ allele and produces C30 but not SafA^FL^. Representative images of the patterns of fluorescence detected for the different strains are shown. The CFP and PC channels are shown. The arrowheads indicate the position of the MCP or MCD forespore poles. The yellow arrows point to partially retractile material which is detected in the strains unable to produce SafA. Scale bar, 1 μm. **B** and **C**: The graphs show the % of cells that display a normal Tgl-CFP localization pattern in the different strains analyzed in **A**. In the majority of the population for the WT (≥ 98%), at hour 6 or 8 of sporulation, Tgl-CFP localizes to the forespore either as 2 caps of fluorescence at the forespore poles (class A), or as a ring of fluorescence around the forespore (class B). These two classes, representing the prevailing Tgl-CFP localization patterns and were scored for sporangia carrying phase dark (panel B) or phase bright spores (panel C). The numbers above the bars correspond to the sum of the two classes. A minimum of 175 cells were scored for each strain over two independent experiments. The mean and standard deviation are represented. **D**: The graph displays the individual forespore/cell fluorescence ratios, normalized by the median value obtained for the wild type strain, grown on the same day (for details see materials and methods section). The median and interquartile range, for the strains analyzed, is also represented. The analysis was only conducted for sporangia with phase bright spores. *n* indicates the number of cells analyzed. The WT values represented are from 8 independent experiments, while the values for the other strains were obtained from 2 independent experiments.

Four Tgl substrates are known, YeeK, GerQ, SafA^FL^, and C30 [31–35]. With the exception of YeeK, which is under σ^K^ control, all other known Tgl substrates are produced early in development, under the control of σ^E^. In previous work we have shown that *safA* is important, but not the exclusive determinant, for the assembly of Tgl [35]. Therefore, we wondered whether other substrates played redundant roles in the assembly of Tgl. To test this possibility, we analyzed the localization pattern of a functional Tgl-CFP fusion in single or multiple mutants unable to produce the known Tgl substrates. The functional Tgl-CFP fusion was previously described; in it, Tgl is separated from the CFP moiety by a 20Å-long, rigid, α-helical linker [35]. In wild-type (WT) sporangia, and in line with previous results [35], Tgl-CFP initially localizes to phase dark spores, as two caps, simultaneously to both the mother cell proximal (MCP) and the mother cell distal (MCD) poles of the engulfed forespore (localization class A); Tgl-CFP then encases the spore forming a continuous ring of fluorescence (class B) [35] (Fig 2A and 2B). Thus, Tgl belongs to class V of coat proteins as defined by McKenney and Eichenberger, which are produced mainly under σ^K^ control and localize to both poles of phase dark spores before encasing the forespore [17]. The accumulation of Tgl at the spore poles, which may correspond to our two-cap (class A) pattern, has been described [37]. Here, and as a reference for the localization of Tgl-CFP in different mutants, the localization of the fusion protein was assessed by scoring sporangia according to classes A and B (as defined above; with one exception, described below, other localization patterns were not considered in this study). Since the main period of *tgl* expression is under σ^K^ control [31, 37–39], we analyzed the localization of Tgl-CFP 6 and 8 hours after sporulation initiation, when σ^K^ is active and the majority of the sporangia have visible phase dark or phase bright spores (Fig 2A). For the WT, classes A and B correspond to ≥ 98% of the sporangia with phase dark or phase bright spores scored, in agreement with previous work [35] (Fig 2B and 2C). In both the *yeeK* and *gerQ* mutants, the sum of classes A and B corresponded to >97% of the sporangia with either phase dark or phase bright spores, similar to the WT (Fig 2B and 2C). Thus, Tgl-CFP is recruited to the forespore surface in nearly all *yeeK* or *gerQ* sporangia. The main difference found for the *yeeK* mutant when compared to the WT was that for sporangia of phase dark spores the representation of class A (49%) and class B (51%) was nearly the same, whereas class A represented 61% of WT sporangia with phase dark spores (Fig 2B). Deletion of *gerQ* also increased the representation of class B in sporangia of phase dark spores (to about 60%). The increase in class B in sporangia of phase dark spores in either mutant suggests that the absence of YeeK or GerQ facilitates encasement by Tgl-CFP. This effect seems mainly mediated by *gerQ*, because in a *yeeK gerQ* double mutant, class B in sporangia of phase dark spores represents 69% (as compared to 62% for the *gerQ* single mutant) (Fig 2A and 2B). These results suggest that YeeK and GerQ, *per se*, have no major impact on the recruitment of Tgl to the forespore but somehow control the encasement step, which seems to be more efficient in their absence. In agreement with the inference that recruitment is largely unaffected in the *gerQ* or *yeeK* mutants, or in the *gerQ yeeK* double mutant, quantification of the forespore/mother cell fluorescence ratio in sporangia of phase bright spores revealed a median value of 1.00 for the WT and *yeeK* strains, a median value of 1.02 for the *gerQ* mutant and of 0.95 for the *yeeK gerQ* double mutant (Fig 2D). Thus, in what concerns the recruitment step, essentially no difference was found between the WT and *yeeK* or *gerQ* mutants, or the double *yeeK gerQ* double mutant.

### Morphogenetic proteins SafA and C30 are the main recruiters of Tgl to the developing spore

We then examined the assembly of Tgl-CFP in a strain where SafA^FL^ is produced, but because the internal Met codons 161 and 164 were changed to Ala codons, the independent production of C30 through internal translation of the *safA* mRNA is eliminated [28, 35]. In this strain, herein termed Δ*C30* (carrying the *safA*^*M161A/M164A*^ allele) the sum of classes A and B still represent the majority (>95%) of the sporangia of phase dark spores, but this number dropped to 89% for sporangia of phase bright spores. C30 thus plays a role in recruitment of Tgl. In agreement with this conclusion, the analysis of the forespore/mother cell fluorescence ratio for the Δ*C30* mutant strain shows that Tgl-CFP accumulates to slightly higher levels in the mother cell (median value of 0.95, compared to 1.00 for the WT strain) (Fig 2D).

In sharp contrast, in the Δ*safA* in frame-deletion mutant, unable to produce both SafA^FL^ and C30, the sum of classes A and B represented only 5% of the sporangia of phase dark spores and 28% of those with phase bright spores (Fig 2A, panels k-q and Fig 2B and 2C). Thus, in the absence of SafA^FL^ and C30, recruitment of Tgl-CFP to the spore surface is severely impaired. Note that in spite of the strong localization defect, the accumulation of Tgl-CFP does not differ significantly from the WT, as shown by immunoblot analysis of whole cell extracts (S1A Fig). Also, that localization improves in sporangia of phase bright spores, further suggests a delay in the assembly of Tgl. In keeping with these inferences, the median value of the forespore/mother cell fluorescence ratio for the Δ*safA* strain (0.69) is considerably lower than for the WT (1.00), implying that the elimination of *safA* causes accumulation of Tgl-CFP in the mother cell (Fig 2D). The Δ*safA* mutation, however, does not completely eliminate the localization of Tgl-CFP, suggesting that YeeK and GerQ may partially compensate for the absence of SafA^FL^ and C30, and that their assembly is partly independent of *safA*. Alternatively, additional, as yet unknown factors, may control the localization of Tgl.

### Partial redundant roles for the Tgl substrates in assembly of the enzyme

We then analyzed the localization of Tgl-CFP in a Δ*safA yeeK gerQ* triple mutant, that is, in the absence of all the known substrates of Tgl (Fig 2A, panels r-b´). In line with the conclusion that SafA^FL^ and C30 are the main factors involved in the recruitment of Tgl, the sum of classes A and B in sporangia of phase dark spores did not differ much from the Δ*safA* single mutant (Fig 2B). Surprisingly, however, in sporangia of phase bright spores, not only the sum of classes A and B represented 45% of the sporangia scored (Fig 2C), but the forespore/cell fluorescence ratio for the triple mutant (0.86) is significantly higher than that of the *safA* mutant (0.69) (Fig 2D). One possible explanation is that the simultaneous absence of SafA, C30, YeeK and GerQ unmasks an alternative SafA-independent pathway that can recruit Tgl-CFP to the spore surface, albeit inefficiently. If so, one possible implication is that additional, as yet unknown Tgl substrates may exist. In previous work we presented evidence indicating that synthesis of the spore cortex peptidoglycan facilitates assembly of Tgl [35].

Finally, we examined the assembly of Tgl-CFP in a strain bearing a *safA* allele with a stop codon replacing the Phe155 codon; in this strain (termed Δ*safA*^FL^, carrying the *safA*^*Phe155STOP*^ allele) C30 is still formed through internal translation but production of SafA^FL^ is eliminated (Fig 2A, panels c’-l’). Tgl-CFP was not detected in sporangia of phase dark spores, and classes A and B represented 16% of the sporangia of phase bright spores, a value lower than that found for the Δ*safA* mutant (Fig 2C). Thus, even though the forespore/mother cell fluorescence ratio of Δ*safA*^FL^ and Δ*safA* sporangia with phase bright spores is very similar (Fig 2D), assembly of Tgl-CFP is more compromised when SafA^FL^ (but not C30) is absent (Δ*safA*^FL^) than when both SafA and C30 are absent (Δ*safA*). Because the localization of C30 is dependent on SafA^FL^ [28, 29] one possible explanation is that C30 retains Tgl-CFP in a mislocalized pattern. Consistent with this supposition, in the majority of the Δ*safA*^FL^ sporangia carrying phase dark spores, Tgl-CFP accumulated as a dot of fluorescence in the MCP forespore pole (Fig 2A, panel c’) (NB: this pattern will be further discussed below). Together, the results suggest that the known Tgl substrates, as well as additional factors, play partially redundant roles in the assembly of the enzyme, with GerQ and YeeK mainly involved in enforcing the normal pattern of encasement, and SafA and C30 primarily involved in recruitment. This partial redundancy in the recruitment of Tgl-CFP to the surface of the developing spore explains why assembly of the enzyme cannot be completely abolished in any of the single mutants studied.

### Non-active site residues at both entrances of the Tgl tunnel are important for enzyme activity

**Fig 3:**
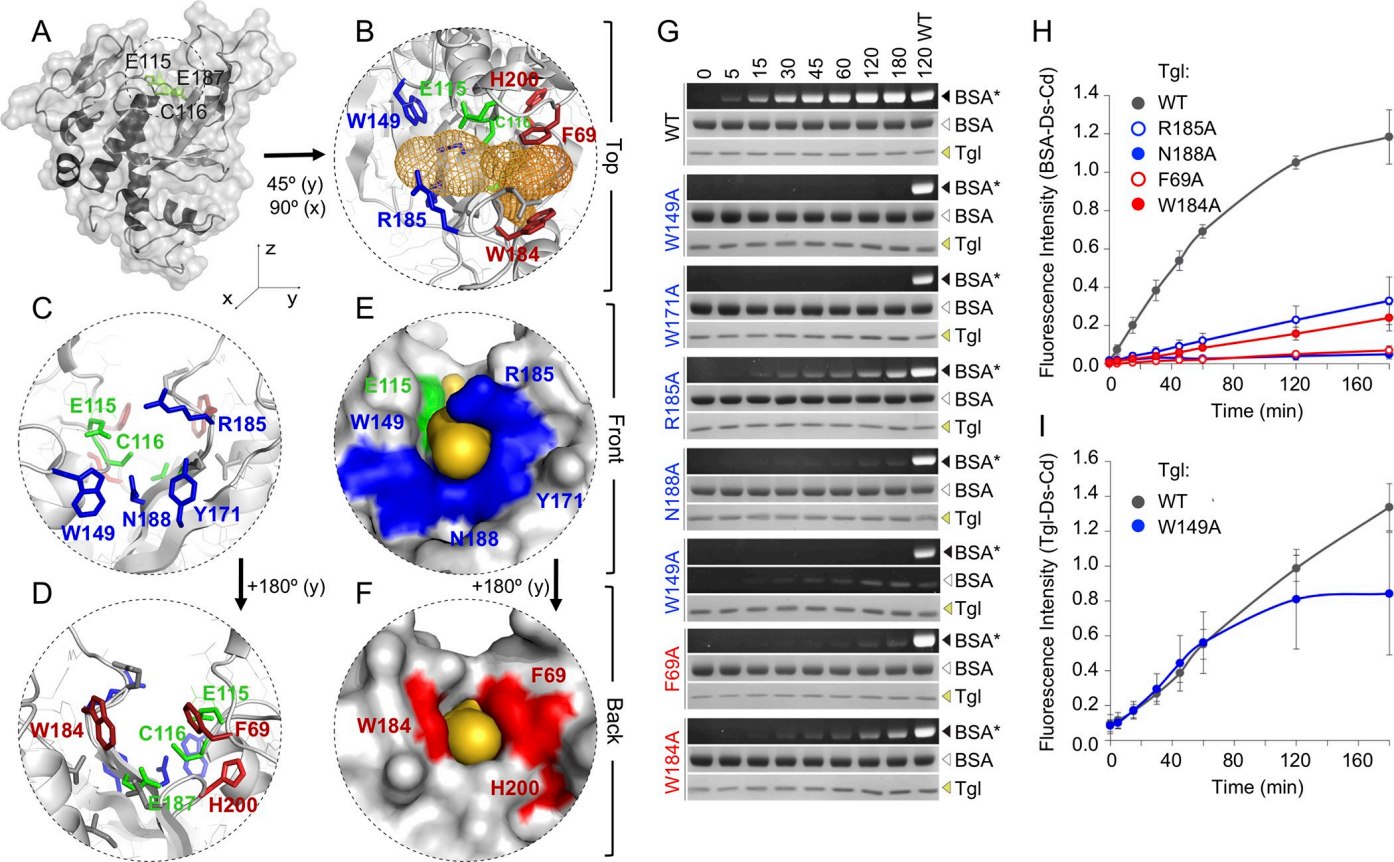
Residues at the front and back entrances of the Tgl tunnel are important for activity. **A**: Cartoon and molecular surface representation of the structure of Tgl. The side chains and surface occupancy of the catalytic residues are shown in green. The active site of Tgl sits in a tunnel that traverses the molecule from side to [14]. **B-F**: Zoomed view of the tunnel region observed from the top (B), front (C, E) or back (D, F). In B and D secondary structure elements and side chains of relevant residues are shown, while in E and F only the surface of the molecule is represented. Relevant residues are highlighted in color: front (in blue), back (red), and catalytic residues (green). The free space of the tunnel is represented in orange, either as a mesh (B) or by surface occupancy (E, F). The models in A-F were drawn using the published structure of Tgl (PDB accession code 4P81). **G**: Tgl (WT type or mutant forms) was incubated at 50°C in the presence of BSA and dansylcadaverine (Ds-cd). Samples were taken at the indicated times (in min) and resolved by SDS-PAGE. The gels were exposed to UV light to reveal labeled BSA (BSA*), and then stained with Coomassie brilliant blue for the visualization of the amount of BSA and Tgl loaded. **H**: shows representative assays for the wild type (WT) enzyme and mutant forms with the indicated single Ala substitutions of residues in the front or back entrances of the Tgl tunnel. **I**: Quantification of the activity of Tgl^W149A^. Note that Tgl^W149A^ did not label BSA, but showed auto-labeling activity (producing Tgl*). Thus, Tgl^W149A^ shows an altered substrate specificity.In **H** and **I**, the fluorescence values obtained for BSA* (**H**) of for Tgl* (**I**) were normalized to the value obtained for the wild type enzyme after 120 min of reaction and plotted for all the assays where fluorescence could be detected (see also [14]).

Since the assembly of Tgl is largely controlled by proteins that are also Tgl substrates, then, it should be possible to identify residues in Tgl, involved in enzyme-substrate interactions, important for the assembly of the enzyme. In addition, mutations in those residues of Tgl could possibly have more pronounced effects on the assembly of the enzyme than the absence of the known substrates individually. Tgl has a distinctive catalytic dyad composed by Cys116 and Glu187, the latter of which can be non-reciprocally substituted by Glu115 [14] (Fig 3A). The catalytic residues of Tgl are located within a tunnel that traverses the molecule from side to side and thus are not surface exposed (Fig 3B–3F). As has been seen for the animal-type TGases, Gln (acceptor) and Lys (donor) proteins that participate in cross-linking reactions (Q and K substrates) approach the active site from opposite sides of a tunnel [40, 41] (Fig 3B). Thus, both entrances of the Tgl tunnel act as substrate docking ports. Accordingly, previous work has shown that His200, located at one of the entrances of the tunnel (in the so-called back side of the enzyme) (Fig 3D and 3F), while not playing an essential role in catalysis, is important for the interaction with substrates and in that way for the overall activity of Tgl [14]. Thus, in searching for additional residues with a role in enzyme-substrate interactions, we focused our attention on four residues at the front entrance of the tunnel (Trp149, Tyr171, Arg185 and Asn188; Fig 3C and 3E), and two residues at its back entrance (Phe69 and Trp184; Fig 3D and 3F). With the exception of Arg185, all of the selected residues are well conserved among Tgl homologues [14]. Arg185 appeared interesting, however, because with other residues in its vicinity, it shows high thermal displacement parameters (B factors) suggesting flexibility [14]. Flexibility in this region of the protein could be important for substrate interactions and for displacement of the ceiling of the tunnel following catalysis, allowing release of a cross-linked product [14] (Fig 3E).

To examine the contribution of the selected residues on the overall activity of Tgl *in vitro*, each was independently substituted by Ala. Tgl^WT^ and its variants were overproduced with a C-terminal His_6_ tag in *E*. *coli* by auto-induction [15]. All the proteins accumulated in *E*. *coli* and we have shown before that the His_6_ tagged-Tgl is functional, both *in vitro* and *in vivo* [15]. The activity of Tgl^WT^ and its variants was then evaluated using BSA as the Q substrate, and the fluorescent primary amine dansylcadaverine as the K substrate. Enzymatic activity can be assessed in this manner by monitoring the formation of fluorescent BSA over time [14]. These assays were conducted at 50ºC, which we showed before to be the optimal temperature for labeling of BSA; presumably, increased conformational flexibility of the substrate makes surface exposed Q residues more prone to labeling [14].

For two of the mutant enzymes tested, Tgl^W149A^ and Tgl^Y171A^, no labeled BSA could be detected (Fig 3G and 3H; not plotted). The activity of Tgl^R185A^ and Tgl^W184A^ was significantly reduced compared to the WT, while Tgl^F69A^ and Tgl^N188A^ showed only residual activity (Fig 3G and 3H). In previous work we showed that no activity could be detected for Tgl^H200A^,Tgl^E115A^ and Tgl^C116A^, while Tgl^E187A^ retained considerable activity (about 60% when compared to Tgl^WT^), as Glu115 acts as a substitute in the absence of the E187 side chain [14]. In all, these results are in line with the view that residues at both entrances of the tunnel are involved in enzyme-substrate interactions and in that way important for the overall activity of Tgl.

### The Q and K sides of Tgl are located at the front and back ends of its tunnel, respectively

While we could not detect accumulation of fluorescent BSA during the time of the amine incorporation assay with the Tgl^W149A^ variant, we detected the accumulation of fluorescent Tgl^W149A^ (Fig 3G). The auto-catalytic activity of Tgl [15] and other TGases has been described [42–46], and during our labeling assay, we could also detect formation of fluorescent Tgl^wt^ concurrently with the labeling of BSA (Fig 3G). Both Tgl^W149A^ and Tgl^wt^ show similar auto-labeling activity during the first 60 minutes of reaction (Fig 3I). Thus, Tgl^W149A^ is essentially functional although it may be somewhat unstable over long incubation times. In any case, and importantly, Tgl^W149A^ shows an altered substrate specificity when compared to Tgl^WT^, as it no longer uses BSA but labels itself. This is in line with the view that residues at the two entrances of the Tgl tunnel, as seen for other TGases, are important for enzyme-substrate interactions (above). More importantly, however, because BSA or Tgl are the Q substrate in the BSA/Tgl-dansylcadaverine labeling reactions, Trp149 must be involved in the interaction with the Q substrate. It follows that the front side of the tunnel, where Trp149 is located, corresponds to the Q substrate docking site, while the back side of Tgl is the site of interaction with K substrates (Fig 3C and 3E).

### Q-side residues are more important than K-side residues for the localization of Tgl

**Fig 4:**
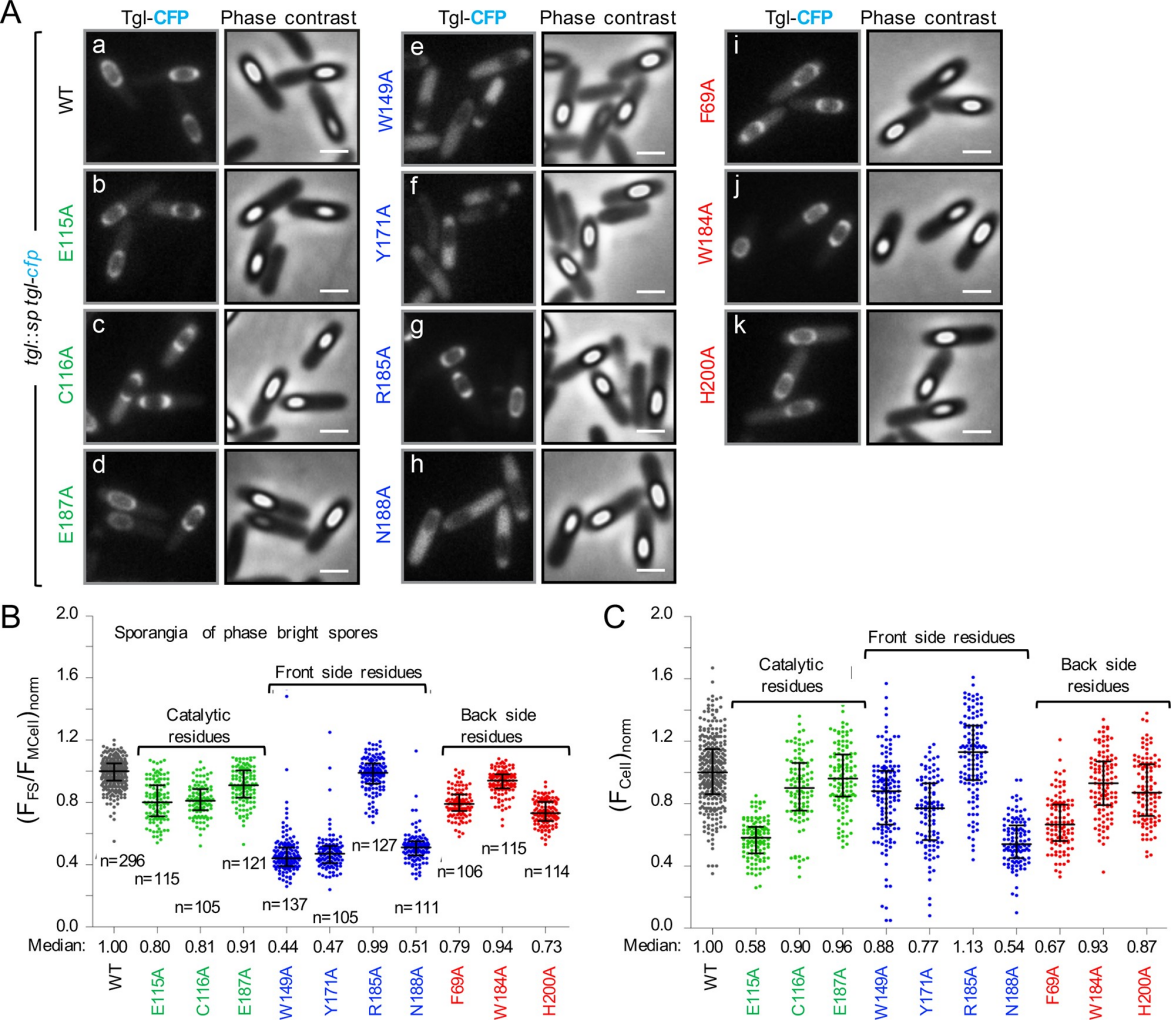
Residues at the front entry of the Tgl tunnel are more important than back residues for the localization of the enzyme. **A**: Forms of Tgl-CFP bearing the indicated single Ala substitutions (color coded according to their role in activity or position in the structure of Tgl; as in Fig 3) were produced from the *amyE* locus in a strain bearing a *tgl*::*sp* insertional allele. Sporulation was induced by resuspension, samples collected 8 hours thereafter, and the assembly of the WT and different forms of Tgl-CFP monitored by phase contrast and fluorescence microscopy. Scale bar, 1 μm. **B**: Cells bearing phase bright spores were analyzed and the normalized forespore/cell fluorescence ratio, (F_FS_/F_cell_)_norm_, was determined (see also Fig 2). For cells of each strain, the individual normalized ratio values, the median, and interquartile range are shown. *n*, number of sporangia scored. **C**: The fluorescence signal detected in whole cells was normalized using the median value of the wild type strain grown on the same day, to give (F_cell_)_norm_ (see materials and methods for details). The same cells were analyzed in **B** and **C**.

Since the substrates of Tgl are important for the localization of the enzyme to the surface of the forespore, Tgl mutants with Ala substitutions of residues located at both entrances of the tunnel should exhibit impaired localization. We tested this prediction by introducing point mutations in *tgl-cfp* and transferring the resulting alleles to the non-essential *amyE* locus of the *tgl*::*sp* insertional mutant. We then analyzed the localization of the different forms of Tgl during sporulation using fluorescence microscopy (Fig 4A). The residues analyzed included not only those located at both entrances of the tunnel that were also tested for a role in enzyme activity (above), but also the residues that form the catalytic center of Tgl, hidden inside the tunnel, that we defined previously [14] (Fig 3).

We determined the forespore/mother cell fluorescence ratio for all strains, producing the WT and mutant forms of Tgl-CFP (Fig 4B). Of the four front side residues analyzed in this way, only the R185A substitution did not seem to impair assembly of Tgl-CFP (median values of the forespore/cell fluorescence ratio of 0.99 and 1.00, for Tgl^R185A^-CFP and Tgl^WT^-CFP, respectively) (Fig 4B). Assembly of Tgl^W149A^, Tgl^Y171A^ and Tgl^N188A^, however, was severely impaired (median values of 0.44, 0.47 and 0.51, for the W149A, Y171A, and N188A substitutions, respectively). Moreover, for these three proteins, fluorescence dispersed throughout the mother cell cytoplasm was seen (Fig 4A, panels e, f, h). Note that all these three residues, W149, Y171 and N188, are located at the Q-acceptor side of the tunnel, and that the surface of N188, in particular, forms the bottom of the front entrance of the tunnel. Forms of Tgl with single Ala substitutions of K-acceptor side (back) residues, Tgl^F69A^, Tgl^W184A^, and Tgl^H200A^, also show impaired assembly, but judging from the quantification of the forespore/mother cell fluorescence ratios, to a lesser degree than front side substitutions (median values between 0.73–0.94) (Fig 4B). Lastly, the assembly of Tgl^C116A^, Tgl^E115A^ and Tgl^E187A^, with Ala substitutions of the catalytic residues buried within the Tgl tunnel, was also reduced as compared to the WT (median values between 0.80–0.91), but to a lesser degree than Q- (median values between 0.44–0.51, R185A excluded) or K-side mutants (median values between 0.73–0.94) (Fig 4C). The catalytic residues are not likely to be directly implicated in substrate docking. Q-donor substrates, however, form an acyl-enzyme intermediate with the catalytic Cys116 residue, in the first step of the reaction catalyzed by TGases. Therefore, while Q-side residues (located at the front entrance of the tunnel) make the most important contribution to the assembly of Tgl, this step may be rapidly followed by the formation of an acyl-enzyme intermediate. In any event, the results suggest that enzyme-substrate interactions are important for the assembly of Tgl. The analysis of the levels of the various forms of Tgl in spore coat extracts is in agreement with the microscopy analysis (S1B Fig; see also the S1 Text).

To test whether the recruitment defects observed for the different forms of Tgl-CFP resulted from protein instability, extracts from sporulating cells were fractionated into a mother cell and a forespore fraction and the presence of the various forms of Tgl assessed by immunoblotting (S1C Fig). We found that all forms of the enzyme showed reduced levels in the forespore fraction, but correspondingly higher levels in the mother cell fraction (S1C Fig; see also the S1 Text). Thus, the mutations did not seem to grossly alter the folding of Tgl. In addition, the total cell fluorescence was measured for the strains expressing the various Tgl-CFP fusions (Fig 4C; see also the material and methods section). Note that we have reported before that the functional Tgl-CFP fusion shows minimal processing and release of the CFP moiety [35]. Some of the point mutations introduced in *tgl-cfp*, for instance E115A and F69A, lead to whole cell fluorescence lower than that found for the WT enzyme. While this analysis may indicate that some of the mutations may slightly alter the folding of Tgl-CFP, we note that there was no direct correlation between the levels at which the different mutant forms of Tgl-CFP accumulate in the cell and their recruitment to the forespore: the total cell fluorescence values were the lowest for Tgl^E115A^-CFP (0.58) and Tgl^F69A^-CFP (0.67), but the recruitment of these forms was not among the most affected, with a median value of forespore/cell fluorescence ratio of ~0.80, while the three lowest values are between 0.44 and 0.51 (Fig 4B and 4C). Thus, the defects observed in the recruitment of the different mutant forms of Tgl-CFP do not seem a direct result of protein instability and likely reflect impaired enzyme-substrate interactions, with Q-side residues (located at the front entrance) making the most important contribution to the assembly of the enzyme.

### Residues important for the localization of Tgl are also important for the cross-linking of C30 in vitro

**Fig 5:**
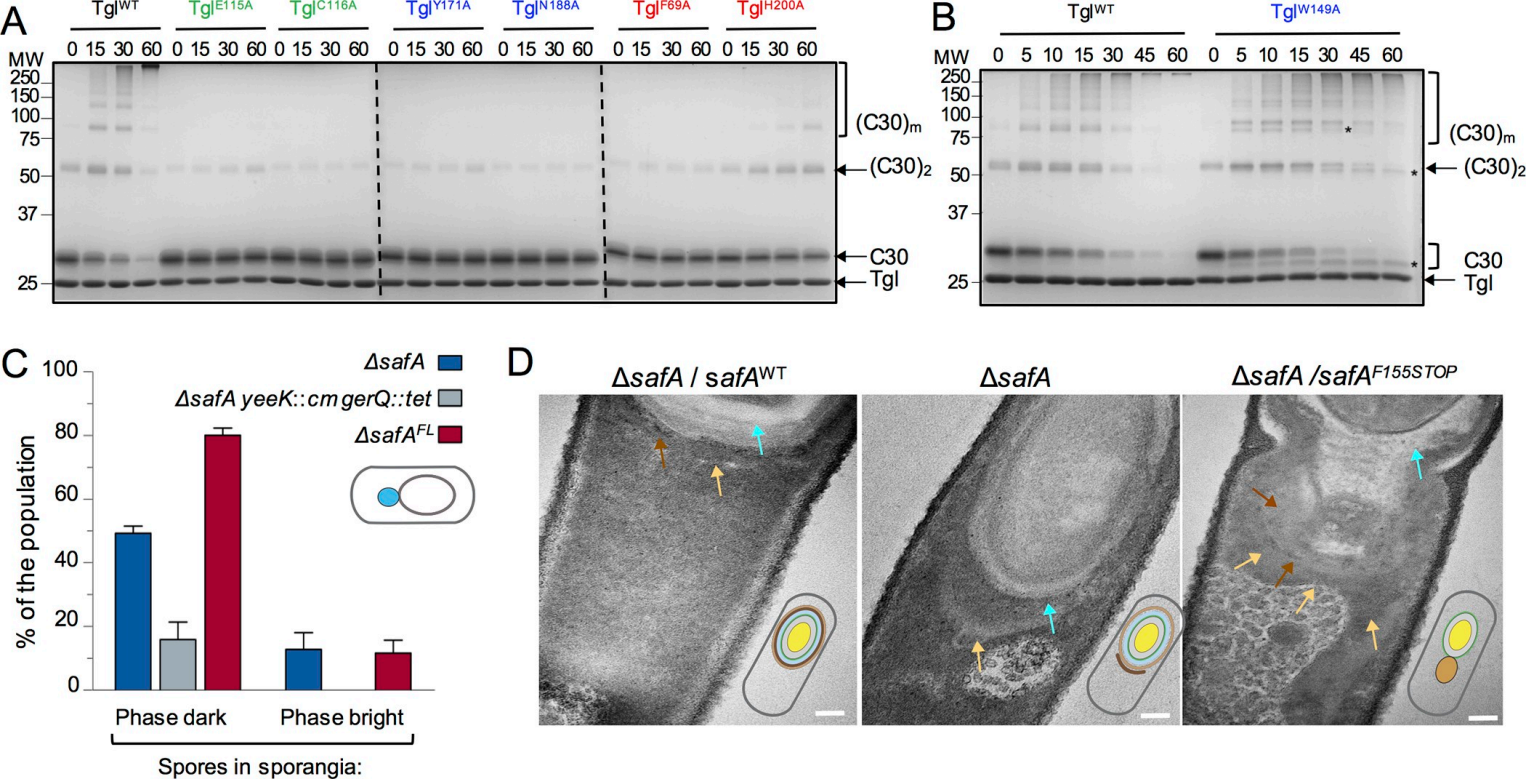
Non-catalytic residues, important for the localization of Tgl are also important for the cross-linking of C30 *in vitro*. C30 and Tgl (either Tgl^WT^ or the indicated mutant forms in **A** and **B**) were incubated at 37°C, and samples were collected at the indicated times (in min). The gels were stained with Coomassie brilliant blue for protein visualization; Tgl and the different forms of C30 are identified on the right-hand side of each panel. (C30)_*2*_ corresponds to a possible dimer of C30, while (C30)_*m*_ represents multimeric forms of C30. The asterisks in panel **B** indicate the site of migration of forms of C30 that are only detected in assays with Tgl^W149A^. Enzymes are color coded according to the location of the residues exchanged relative to the tunnel of Tgl: green, catalytic residues; blue, front side of the tunnel; red, back side of the tunnel. The site of migration of the molecular weight markers, in kDa, is indicated on the left side of each panel. **C**: Production of C30 in the absence of SafA^FL^ causes mislocalization of Tgl-CFP. Tgl-CFP was produced in the indicated strains and the percentage of sporangia in which the fusion protein localized as a single dot of fluorescence on the MCP pole of the forespore (schematically represented on the right side) was scored. Only strains where this pattern of localization was observed are illustrated. Note that the Δ*safA*^*FL*^ mutant bears the *safA*^*F155STOP*^ allele, and only produces C30 (see text for details). The mean and standard deviation are represented. **D**: Samples were collected from sporulating cultures of the indicated strains 6 hours after the onset of the process and analyzed by thin sectioning TEM. Note that the Δ*safA* allele was complemented at *amyE* with either *safA*^wt^ or *safA*^*F155STOP*^. The blue arrows points to the cortex region, the brown arrows to inner coat material and the yellow arrows to the position of outer coat material. Scale bar, 100 nm.

One prediction arising from the results described in the preceding section is that at least some of the Ala-substituted forms of Tgl should show reduced cross-linking activity towards its natural substrates. We used C30 to test this prediction as this is the substrate whose absence, *per se*, more severely impairs assembly of Tgl (above). Thus, the cross-linking activity of Tgl mutant forms whose recruitment appears more compromised, with either Ala substitutions of residues at the Q and K entrances of the tunnel, were analyzed in parallel with Tgl^WT^ and two variants with Ala substitutions of catalytic residues. C30 and Tgl (WT and mutant forms) were independently over-produced in *E*. *coli* with a C-terminal His_6_-tag, and the various proteins were purified and incubated together at 37°C. This is the temperature at which the microscopy experiments were conducted; note, however, that the optimal temperature for Tgl activity is 50°C [14, 15], and it was at this temperature that the BSA labeling assays were conducted (see also above). A cross-linking assay with Tgl and C30 or BSA as the substrates, conducted at 50º is shown in S3 Fig, for reference. Tgl^WT^ efficiently cross-linked C30 into high molecular weight species, C30_*m*_ (Fig 5A and 5B, parenthesis). For Tgl^E115A^, Tgl^C116A^, Tgl^Y171A^ and Tgl^N188A^, no activity could be detected [Fig 5A; note that the C30 species presumed to be a dimer, (C30)_*2*_, resistant to the SDS-PAGE conditions, is detected in all assays at time 0]. In contrast, Tgl^H200A^ displayed significant activity when compared to the other forms of the enzyme, with the formation of high molecular weight products during the time of the assay (Fig 5A). Tgl^W149A^ displays significant enzymatic activity but an altered specificity: it shows auto-labeling activity with dansylcadaverine but is unable to label BSA (Fig 3G and 3I; see above). Yet, our results also show that the assembly of Tgl^W149A^ is compromised (Fig 4 and S1B Fig). The C30 cross-linking assays, however, show that Tgl^W149A^ produces cross-linked species that are not detected with Tgl^wt^ or any of the other mutant forms of the enzyme (Fig 5A and 5B, asterisks). While these forms may be the result of intramolecular cross-linking, the observation shows that Tgl^W149A^ interacts with C30 differently from Tgl^WT^. Presumably, then, altered interaction of Tgl^W149A^ with C30 does not permit efficient recruitment of the enzyme *in vivo*.

### C30 retains Tgl^WT^, but not forms of the enzyme with single Ala substitutions of Q- and K-side residues, in the mother cell

In the three mutants, Δ*safA*, Δ*safA*^FL^ and the triple mutant Δ*safA*, *yeeK gerQA*, in which the class A/class B pattern of Tgl-CFP localization is more significantly reduced (quantification in Fig 2B–2D), Tgl-CFP is detected in the majority of sporangia with either phase dark or phase bright spores as a strong dot of fluorescence at the MCP pole of the forespore (Fig 2A). Quantification of the dot pattern for the three strains shows a dot in 80% of the sporangia with phase dark spores and 12% of those with phase bright spores for the Δ*safA*^FL^ mutant (Fig 5C). The dot pattern was less represented in the Δ*safA* single mutant or the *yeeK gerQ* Δ*safA* triple mutant (Fig 5C). We used transmission electron microscopy (TEM) to characterize further the phenotype of the Δ*safA*^FL^ mutant (Fig 5D). The TEM images of sporangia at an intermediate stage of sporulation shows the accumulation of coat material at the MCP forespore pole in Δ*safA* sporangia and projecting into the mother cell cytoplasm, as previously noted [22] (Fig 5D, middle panel). This phenotype is corrected by *safA*^wt^ at *amyE* (Fig 5D, left panel). Strikingly, accumulation of coat material was seen at the MCP forespore pole in Δ*safA*^FL^ sporangia (Fig 5D, right panel). In the Δ*safA*^FL^ strain, the C30 form, but not SafA^FL^, is produced [28]. Because C30 is not assembled in the absence of SafA^FL^ [27, 28], one possibility is that C30 retains Tgl-CFP, along with other coat proteins, in the mother cell.

**Fig 6:**
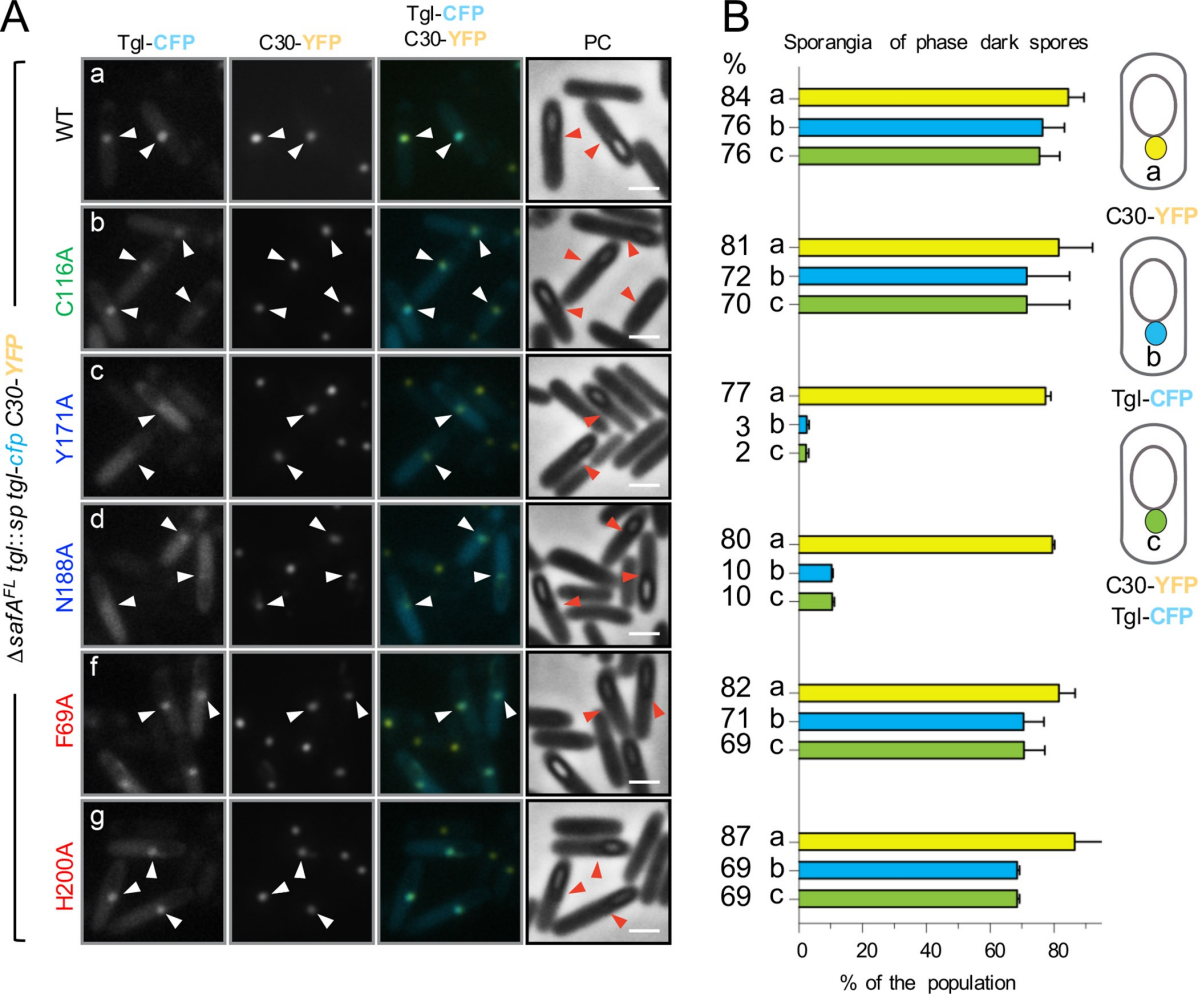
C30 retains Tgl^WT^-CFP but not front side mutants in the mother cell cytoplasm. **A**: Forms of Tgl-CFP bearing the indicated single Ala substitutions (color coded according to their role in activity or position in the structure of Tgl; as in Fig 3) were produced from the *amyE* locus in Δ*safA*^FL^ strain bearing a *tgl*::*sp* insertional allele and C30-YFP produced from the *thrC* locus. Sporulation was induced by resuspension, samples collected 8 hours thereafter, and the localization of the CFP or YFP fusions monitored by phase contrast (PC) and fluorescence microscopy. Scale bar, 1 μm. White arrowheads indicate the position of the fluorescence signal from Tgl-CFP or C30-YFP in cells in which a forespore could be detected (red color is used for the phase contrast images). Scale bar, 1 μm. **B.** The graphs, aligned with the panels in A, show the percentage of sporangia with phase dark spores that showed C30-YFP localized as a dot of fluorescence in the MCP pole (class a) and Tgl-CFP with a similar pattern (class b). Sporangia were also scored for the co-localization of C30-YFP and Tgl-CFP at the MCP pole of the forespore (class c). A diagram of each localization class is shown on the right side of the panel. The graphs display the mean and standard deviation for each class. The numbers on the left side indicate the mean value obtained for each class in each strain. A minimum of 200 cells were scored for each strain in each of two independent experiments.

If so, Tgl^WT^ and C30 should co-localize in Δ*safA*^FL^ sporangia. Conversely, Tgl variants whose assembly and the ability to cross-link C30 *in vitro* is impaired should not co-localize with C30. To test these predictions, we co-expressed *C30-yfp* along with *tgl*^*wt*^-*cfp* in the Δ*safA*^FL^ mutant. Because the single fluorescence dot pattern of Tgl-CFP is more easily seen in sporangia with phase dark spores, the co-localization of Tgl-CFP and C30-YFP was only scored in these cells. We found that C30-YFP localizes as a bright dot of fluorescence in the majority of the sporangia (84%) and that Tgl^wt^-CFP co-localized with C30 (in 76% of the sporangia scored) (Fig 6A and 6B). Forms of Tgl with Ala substitutions of K-side residues (F69A, H200A), or the catalytically inactive Tgl^C116A^-CFP form, also co-localized with C30-YFP (values varying between 69 and 70%, as indicated in Fig 6B), although a weak CFP fluorescence signal was also seen dispersed throughout the mother cell cytoplasm (Fig 6A). Strikingly, however, Tgl^Y171A^-CFP and Tgl^N188A^-CFP (with Ala substitutions in Q-side residues) largely accumulate throughout the mother cell cytoplasm, and only co-localize with C30-YFP in 2 and 10% of the sporangia scored (Fig 6A and 6B). Note, however, that the C30-YFP dot at the MCP forespore pole is maintained in *tgl*^Y171A^-*cfp* (77% of the sporangia) or *tgl*^N188A^-*cfp* (80%) sporangia. Thus, forms of Tgl with Ala substitutions of residues in the Q acceptor side of Tgl, which show impaired assembly, but much less so K-side residues, fail to co-localize with C30 in the mother cell cytoplasm. This observation lends strong support to the idea that assembly of Tgl is mainly determined by its interaction with the SafA^FL^/C30 substrates and that Q-side residues make a more important contribution to the assembly of Tgl than do K-side residues.

As a control for these experiments, we also wanted to test whether a SafA-dependent protein that is not a direct substrate of Tgl would still co-localize with C30 when the cells produce a form of Tgl, such as Tgl^Y171A^ whose localization is impaired. Since in the absence of SafA^FL^ accumulation of some coat material is seen by TEM at the MCP forespore pole (Fig 5D; see above), we expected that such a protein would form dots at the MCP forespore pole regardless of the form of Tgl produced by the cells. A YaaH-GFP fusion was shown before to be dependent on SafA for localization to the inner coat [17, 25, 36] and YaaH is not a Tgl substrate [30–35]. We constructed a YaaH-YFP fusion and examined its localization as well as the localization of Tgl^WT^-CFP or Tgl^Y171A^-CFP, in the strain producing C30 but not SafA^FL^ (S4A Fig). In this experiment, Tgl-CFP formed a dot in 88% of the sporangia examined, while dots were only seen in 5% of the sporangia for Tgl^Y171A^, the most common pattern being the fluorescence signal dispersed throughout the mother cell cytoplasm (S4B Fig, class b; see also above). For YaaH-YFP, however, no dots of fluorescence were seen at the MCP forespore pole; instead, YaaH-YFP localized as a ring around the forespore (S4A and S4B Fig). This result shows that mislocalization of C30 is not sufficient to retain all of the known SafA-dependent proteins in the mother cell. On the other hand, it strongly reinforces the specificity of the Tgl^WT^-C30 interaction and its requirement for the proper localization of Tgl.

### Reaction of Tgl with the C30 substrate

**Fig 7:**
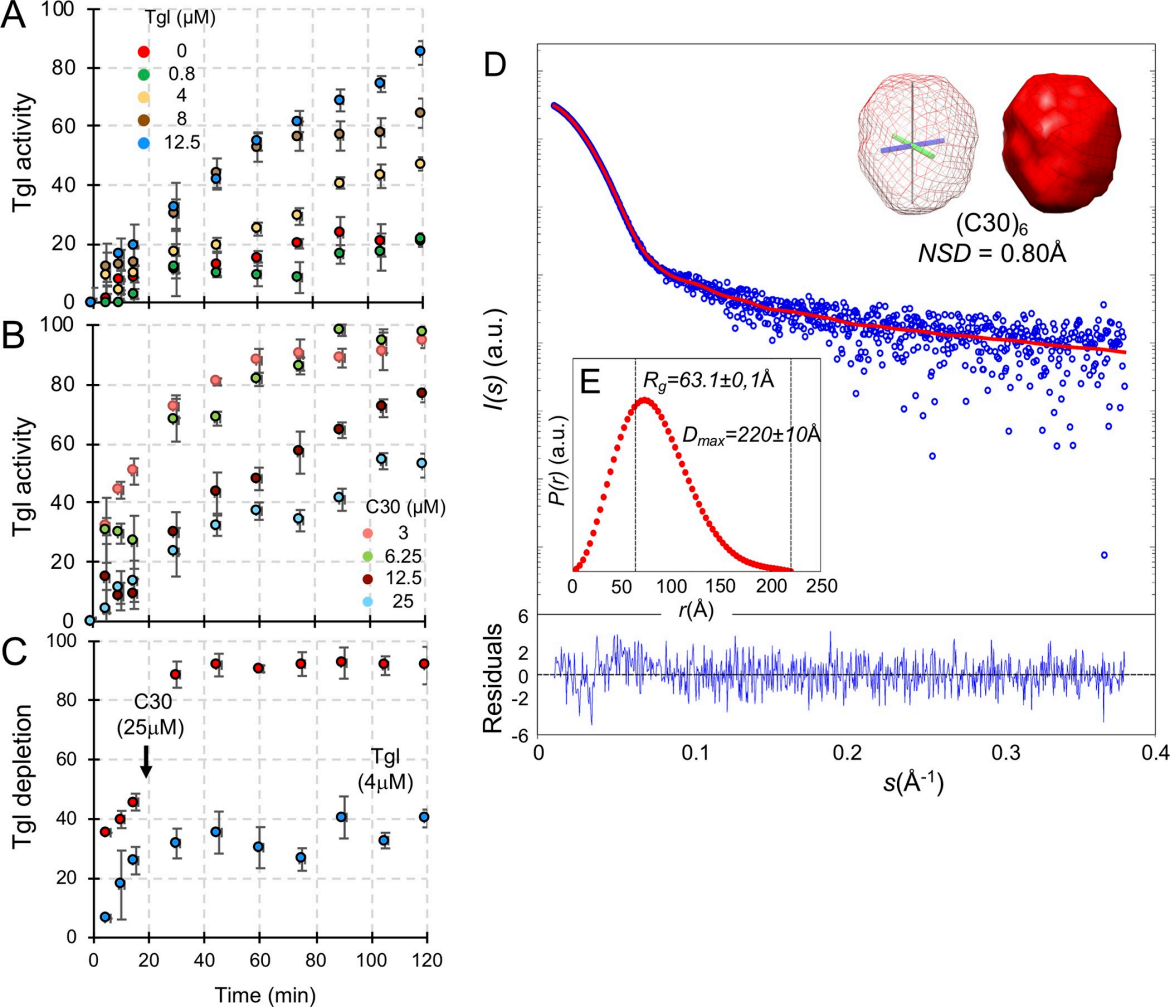
Reaction of Tgl with C30 and *ab initio* model of C30. **A**: Tgl, at the indicated concentrations, was incubated with purified C30 (at 25 μM; see also S6A Fig) and samples of the reaction mixture collected at the indicated times. The samples were mixed with loading dye and boiled to stop the reaction and resolved by SDS-PAGE. The level of C30 decreases over time as the result of Tgl-mediated cross-linking into higher molecular weight forms (see S5A Fig for representative SDS-PAGE gels). Tgl activity is expressed as the level of C30 in each time point, normalized to the initial amount of the protein. **B:** The assays were conducted as described in A, except that Tgl (at 8 μM) was incubated with different concentrations of C30, as indicated (see S5B Fig for representative SDS-PAGE gels). **C**: Tgl (at 4 μM) was incubated alone during 20 min and C30 added at this point, to a final concentration of 25 μM. Samples were taken along time and analyzed by SDS-PAGE (representative gels are shown in S5C Fig). The level of Tgl in each sample was quantified and normalized to the initial amount of protein. In A-C, experiments were repeated three times with different preparations of Tgl and C30. **D**: SAXS intensity of C30 (blue circles), *I(s)*, is represented in logarithmic scale as a function of the momentum of transfer, *s*. The inset shows the SAXS-generated *ab initio* molecular envelope obtained by clustering and averaging models from 20 independent runs, with a Normalized Spatial Discrepancy (NSD) of 0.8 Å. The low-resolution reconstruction of C30 in solution is compatible with a high-order macromolecular assembly, most likely a hexamer, termed (C30)_6_. Both mesh and solid representations of the model are shown, both centered at the origin of the cartesian axis. The red line corresponds to the scattering profile calculated from the *ab initio* model that best fitted the experimental data (χ^2^ = 2.2). Point-by-point residuals of the fitting and the absolute intensities are shown in the bottom panel. **E**: Pair-wise distance distribution (*P(r)*) of SEC-purified C30 calculated from data in the range 0.010<*s*< 0.38Å^-1^ (see the Material and Methods for details). The derived *R*_*g*_ and *D*_*max*_ values are displayed in dashed lines.

The results in the preceding sections suggest a model in which Tgl is recruited to the surface of the developing spore by its pre-assembled substrates, with SafA and C30 making the most important contribution. This model raises the possibility that the activity of Tgl is itself controlled by the local concentration of the pre-assembled substrates. To investigate how the activity of Tgl varied with the concentration of its substrates, we conducted cross-linking assays using purified C30. In a first series of assays, C30, at a fixed concentration (25 μM) was incubated with various concentrations of Tgl, and the formation of cross-linked species monitored by SDS-PAGE. Total cross-linking was estimated by measuring the decrease in the representation of C30 over time, which is converted into high molecular weight forms (S5A Fig; see the material and methods for details on the quantification). Incubation of C30 alone results in the formation of the (C30)_2_, dimer (at about 60 kDa) that resists the denaturing conditions of SDS-PAGE (S5A Fig). Formation of this species was increased for long incubation times in the presence of the lowest concentration of Tgl tested (0.8 μM); at an intermediate concentration of Tgl (4 μM), the cross-linking activity increased linearly with time (Fig 7A). For the next concentration of enzyme tested (8 μM), activity increased linearly during the first 70 min of incubation but then reached a plateau (Fig 7A). For a higher concentration of Tgl (12.5 μM), however, activity increased linearly up to 120 minutes of incubation (Fig 7A). Together, these results suggest that the enzyme is somehow inactivated over time, in a manner that depends on the enzyme/substrate ratio.

If so, then the plateau effect seen at 8 μM of Tgl should be eliminated by decreasing the concentration of substrate. To test this, Tgl, at a final concentration of 8 μM, was incubated with progressively lower concentration of C30. Surprisingly, we found that the activity of Tgl decreased with an increase in substrate concentration (Fig 7B; representative gels are shown in S5B Fig). When C30 was present at 25 μM, the highest substrate concentration tested, enzyme activity also reached a plateau, after 60 min of incubation (Fig 7B). Inspection of the SDS-PAGE gels used to quantify the activity of Tgl show that the enzyme is depleted over time, in a manner that is dependent on the concentration of substrate (S5A and S5B Fig). This suggests that Tgl itself becomes cross-linked. To more precisely test this idea, we incubated Tgl alone (at 4 μM) and after 20 min, C30 was added to a final concentration of 25 μM. During the first 20 min of the reaction, the intensity of the band corresponding to Tgl, at 26 kDa, is slightly reduced (S5C Fig). When C30 is added (to 25 μM), however, the intensity of the Tgl band is rapidly reduced, concomitantly with the formation of the (C30)_2_ dimer (S5C Fig). The quantification of this effect is shown in Fig 7C. The decrease in Tgl when incubated alone is consistent with the previously described auto-cross-linking activity [14]) and with the auto-labelling activity detected in the presence of dansylcadaverine (above). Together, these results suggest that Tgl itself is cross-linked during the reaction, and that the ratio of Tgl/substrate will limit the duration and extent of C30 cross-linking.

### C30 is an oblong hexamer in solution

C30 self-interacts in a yeast two-hybrid system [27], and it has been proposed that it forms high molecular weight complexes that are cross-linked by Tgl at the surface of the developing spore [35]. The oligomeric state of C30 in solution, however, is not know. Also, the cross-linking assays described in the preceding section indicate that Tgl is itself cross-linked to C30, which could possibly be facilitated if C30 forms an oligomeric structure. To gain insight into the oligomeric state of C30, the purified protein (S6A Fig) was analyzed by size exclusion chromatography (SEC) and small-angle X-ray scattering (SAXS). In SEC, purified C30 eluted as a main single sharp peak (*ca* 1 ml wide) corresponding to a molecular mass of 151.4 ± 7.5 kDa, *i*.*e*., the mass of a possible hexamer, herein termed (C30)_6_ (S6B Fig). The peak fractions were then analyzed by SEC-SAXS [47]. SAXS data indicate that (C30)_6_ is a globular particle in solution with a radius of gyration, *R*_*g*_ of 63.1 ± 0.1 Å and a maximum intramolecular distance, *D*_*max*_ of 220.0 ± 10.0 Å (Fig 7C). The molecular weight estimation from the SAXS data is of 160.1 ± 15 kDa, in good agreement with the SEC data, and suggesting that in the absence of Tgl the particle is a stable hexamer. The C30 oligomer in solution can accommodate six molecules of Chymotrypsinogen A, a monomeric globular protein with a molecular mass similar to a C30 monomer (25.9 kDa) ([48]; S6C Fig). The smooth asymmetrical pair-wise distance distribution, *P(r)*, indicates that (C30)_6_ is slightly aspheric (Fig 7E). The corresponding low-resolution shapes of (C30)_6_ reconstructed based on the *P(r)* [47] (inset in Fig 7D) fit the experimental data well (χ^2^ = 2.2), and provide an oblong envelope model (average NSD = 0.8; see also the material and methods section).

This analysis suggests that C30 forms a large oligomer, whose repeating unit may be the (C30)_6_ hexamer detected both in our SEC and SAXS analysis. C30 is detected as a dimer by SDS-PAGE even in the absence of Tgl (Figs 5A, 5B, 5A and S5B) suggesting that this species is highly stable. In the presence of Tgl, (C30)_2_ increases rapidly before higher molecular weight forms of the protein accumulate (Fig 5A and S5 Fig; see also above). This suggests that (C30)_6_ is a trimer of cross-linked dimers, although other architectures can explain the accumulation of cross-linked (C30)_2_. A cross-linked (C30)_6_ hexamer in turn, may be an intermediate in the formation of higher molecular weight species. The size of the complexes obtained in the presence of Tgl precluded, for the moment, a study by SAXS. In any event, we posit that (C30)_6_ and higher order oligomers accumulate at the surface of the developing spore, and that these pre-assembled complexes recruit Tgl. Cross-linking of these species by Tgl may be self-limiting, in that the enzyme becomes cross-linked into the forming structure, and thereby unable to conduct further catalysis.

## Discussion

We show that the recruitment of Tgl to the forming coat is largely controlled by its substrates, in what we refer to as substrate-driven localization. Of the four known Tgl substrates, SafA^FL^ and C30 are the main factors controlling recruitment of Tgl, whereas YeeK and GerQ appear to act mainly to delay encasement. Encasement is controlled by the SpoVID protein, with which SafA directly interacts [49, 50]. Thus, spore encasement by Tgl may rely primarily on its interaction with SafA^FL^ and C30, and it seems plausible that GerQ and YeeK compete with Tgl for a binding interface in SafA, or that in their absence SafA is more accessible to Tgl.

While the independent formation of C30 through internal translation is not essential for inner coat formation, the corresponding region in SafA^FL^, *i*.*e*., the C-terminal moiety of the protein (Fig 1B), is likely to have the main role in inner coat assembly. Consistent with the idea that the C30 region carries the morphogenetic information for inner coat assembly, not only does the overproduction of C30 in an otherwise WT background blocks sporulation [28] but, as we now show, production of C30 in the absence of SafA^FL^ results in the retention of Tgl and the accumulation of coat material at the MCP forespore pole (Fig 5D and Fig 6). Deletion of *safA* causes a much less severe coat mislocalization phenotype [27, 36]. Possibly, only inner coat material is misassembled in a *safA* mutant, whereas C30 additionally retains outer coat material in the mother cell (Fig 5D). Nevertheless, since YaaH-YFP still forms a ring around the forespore not all inner coat proteins are retained in the mother cell when C30 is produced in the absence of SafA^FL^ (S4 Fig). In any event, the retention of Tgl^WT^ by C30 at the MCP forespore pole, but not of Tgl variants with Ala substitutions of Q-side residues (Fig 6), provides strong support for the substrate-driven localization model.

**Fig 8:**
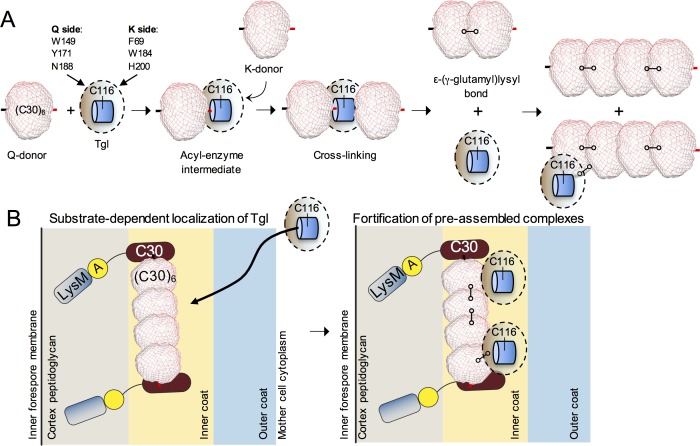
Control of Tgl localization and activity at the spore surface. **A**: reactions of (C30)_6_ with Tgl. We propose that the (C30)_6_ hexamer acts both as a Q and K donor and becomes cross-linked by Tgl to form a dimer of hexamers. An intermediate step in this reaction (not represented for simplicity) appears to be the formation of cross-linked dimers within the (C30)_6_ hexamer, which may be a trimer of cross-linked dimers (see the Discussion for details). With time, this species becomes cross-linked into larger cross-linked oligomers, in what may represent *de novo* protein polymerization. Tgl itself is cross-linked into the forming structure. **B**: SafA is recruited early to the surface of the developing spore, prior to synthesis of Tgl. At the spore surface, SafA is found in at least two forms: SafA^FL^ (full-length) and C30. SafA^FL^ has a localization signal formed by a LysM domain and region A [30]. The protein is found in association with both the cortex peptidoglycan and in the inner coat [30] but only the first localization is depicted for simplicity. C30 interacts with SafA^FL^ via the corresponding region in the latter. A likely interaction of SafA^FL^ with itself is not represented. As C30 lacks localization signals, this interaction is required for the localization of C30 to the coat. SafA^FL^ and C30 are key determinants in the recruitment of Tgl to the coat (left panel), placing the enzyme in close proximity to its substrates. Residues involved in substrate recognition, mainly in the Q side of the enzyme (light brown shading), are required for the localization of Tgl. In a second step, Tgl cross-links C30 (and presumably C30 to the corresponding region of SafA^FL^; not represented) to fortify pre-assembled complexes of SafA^FL^ and C30. During the process, Tgl itself also becomes cross-linked, which may eventually limit its activity at the spore surface. The proteins are not drawn to scale.

C30 self-interacts, C30 interacts with SafA^FL^ and the latter interacts with itself via the C30 region [27–29]. It has been suggested that these interactions create a multivalent platform for binding of the inner coat proteins and that Tgl exerts mainly a “spotwelding” activity, fortifying pre-formed assemblies rather than catalyzing *de novo* protein polymerization [35]. Both types of activities, however, have been described for human and human-like TGases ([2]; see also below). Our finding that C30 is a hexamer in solution (Fig 7 and S6 Fig) provides strong support for both ideas (Fig 8). In addition, the Tgl-mediated cross-linking of (C30)_6_ into larger molecular weight species in vitro may represent a case of *de novo* protein polymerization. In any event, it seems likely that Tgl cross-links and fortifies *in vivo* of a scaffold formed through the interactions among the various forms of SafA (Fig 8).

The requirement for its substrates for the localization of Tgl then appears as a mechanism for directing the enzyme to the exact locations, within the coat, where its “spotwelding” activity is needed. SafA^FL^ recruits C30, YeeK and GerQ to the inner regions of the coat [51]. Tgl itself is recruited to the cortex region and to the inner coat by SafA and we argued before that it most likely acts to cement the cortex/inner coat interface [27, 29, 35]. As we recently showed, SafA itself has an important role in proper assembly of the cortex/inner coat and inner/outer coat interfaces [30]. Since at least SafA^FL^, C30 and GerQ are produced earlier than Tgl, recruitment of the enzyme is governed by its time of synthesis, which is primarily under σ^K^ control. Thus, substrate-driven localization imparts both spatial and temporal control over the activity of Tgl, positioning the enzyme in the vicinity of its substrates, so that the correct proteins or assemblies are timely cross-linked, reminiscent of the “assembly-driven regulation of cross-linking” described for FXIII [2, 5, 52]. Incubation of Tgl with C30, while leading to cross-linking of C30, also brings about cross-linking of Tgl, in a manner that is influenced by the concentration of substrate (Fig 7A–7C and S5 Fig). Since Tgl is found as high molecular weight cross-linked species in both the cortex and inner coat of mature spores [35], it is tempting to speculate that Tgl is eventually immobilized through cross-linking (Fig 8), limiting its action during maturation of the coat.

The substrate-dependent localization of Tgl may also minimize unwanted protein cross-linking reactions. The crystal structure of Tgl shows the catalytic center of the enzyme located within a 15Å-long and ~6Å wide (on average) tunnel that crosses the molecule [14]. While the tunnel appears narrow, and Tgl may be a narrow-specificity enzyme, it is able to catalyze amine incorporation into and cross-link of non-physiological substrates such as BSA (Fig 3G and S3 Fig), especially at high enzyme/substrate concentrations, conditions that conceivably could also be found in the coat [14, 15].

Residues at the two entries of the tunnel are the docking sites for the Q and K substrates [14] (Fig 3). We present evidence suggesting that the Q-substrate docking site of the enzyme corresponds to its front side, where Trp149 is located, and we show that front (Q) side residues make more important contributions to the localization of Tgl than back (K) side residues (Fig 5A and 5B). Following binding to the enzyme, the Q- substrate forms a γ-glutamythiolester with the active site Cys116, a step that is also dependent on Glu187 or Glu115 [14]. The K-substrate then binds the acyl-enzyme intermediate and attacks the thiolester bond, re-generating the active site Cys 116 residue and resulting in protein cross-linking (Fig 1B). Formation of the acyl-enzyme intermediate is thought to be the rate-limiting step in the reactions catalyzed by TGases [2]. During coat formation, however, docking of the Q-substrate, appears to be the limiting step in the localization of Tgl (Fig 4; see also the final model in Fig 8). After this interaction, a second Tgl-substrate interaction is allowed at the back-side entrance of the tunnel, with the K substrate. That back-side residues do not compensate for Ala substitutions of front side residues, is in line with the K substrate interaction only occurring after docking of the Q-substrate and formation of the acyl-enzyme intermediate as proposed for the reaction cycle of TGases (Fig 1B). It seems possible that the K-substrate interaction does not immediately follows Q-substrate docking, as the cross-linking activity of Tgl is delayed until spore release [33, 53]. Although Tgl cross-links itself in the cortex and inner coat [35], as mentioned above, it is unlikely that the activity of Tgl is directly required for its localization. Not only the buried catalytic residues make a smaller contribution than Q- or K-side residues but Tgl^C116A^, inactive in vitro, is less impaired for localization than Tgl^H200A^, which is active in vitro (Fig 3 and Fig 5A and 5B). This is consistent with the idea that enzyme-substrate docking is the main mechanism controlling the localization of Tgl, with formation of the acyl-enzyme intermediate stabilizing the Q-substrate-enzyme interaction. With respect to C30, (C30)_6_ acts as both the Q and K donor substrate (Fig 8).

The substrate-dependent localization mechanism that we propose for Tgl is also akin to that of some PBPs, the localization of which is prevented by active site mutations, substrate reduction or masking by certain antibiotics, in that it efficiently couples sub-cellular localization and proper timing of enzyme activity [54–57]. It differs in that no substrates other than the terminal D-Alanyl-D-Ala of the peptidoglycan stem peptide are known for the PBPs. TGases, in contrast, have the potential to cross-link proteins other than their cognate substrates, and the time and site where they exert their activity is tightly regulated, as exemplified for FXIIIa. All known human and human-type TGases undergo complex activation mechanisms, and even another bacterial TGase, the MTG protein from *Streptomyces mobaraensis*, which is structurally characterized, is produced as an inactive zymogen [2–4, 58, 59]. In contrast, Tgl is synthesized in active form. The substrate-dependent localization of Tgl may thus the primary mechanism to ensure proper temporal and spatial control of spore coat protein cross-linking, as well as specificity and the extent of its action (Fig 8). Substrate-dependent localization may similarly control the activity of Tgl orthologues in other spore-forming bacteria.

## Material and methods

### Bacterial strains, media and general methods

The construction of all plasmids and strains is described in the supporting material; strains, plasmids and primers used in this study are listed in S1, S2 and S3 Tables.

### Fluorescence microscopy

Sporulation was induced by the resuspension method as described [60]. Briefly, cultures were grown in growth medium, and the cells collected at an OD_600_ of ~0.4, and transferred to a minimal medium in which sporulation is induced. Samples were taken 6 and 8 hours after resuspension (defined as the onset of sporulation), the cells collected by centrifugation (1 min at 2.400 x g, room temperature), and washed with 1 ml of phosphate-buffered saline (PBS). Finally, the cells were resuspended in 20 μl of PBS and applied to microscopy slides coated with a film of 1.7% agarose. Images were taken with standard phase contrast, CFP and YFP filters, using a Leica DM 6000B microscope equipped with an aniXon+EM camera (Andor Technologies), and driven by Metamorph software (Meta Imaging series 7.7, Molecular Devices).

### Image analysis

Image analysis was conducted with Meta Imaging series 7.7 software and the determination of the values of fluorescence in the forespore or in the mother cell cytoplasm was conducted as described [35]. The individual values of the forespore/cell fluorescence ratio, (F_FS_/F_cell_), or cell fluorescence, F_cell_, were examined with GraphPad Prism 5 (GraphPad Software, Inc) which indicated that the values showed a non-normal distribution. Thus, for normalization purposes each individual value was divided by the median value of the wild type strain that had been grown on the same day and under the same conditions; the same normalization was conducted for the individual values of the wild type strain. The calculation of the normalized values of the forespore/cell fluorescence ratio, (F_FS_/F_cell_)_norm_, or cell fluorescence, (F_cell_)_norm_, was according to Eqs 1 and 2, respectively:

$${\left(F_{FS}/F_{cell}\right)}_{norm}=\frac{\left(F_{\mathrm{FS}}/F_{\mathrm{cell}}\right)}{\mathrm{median}\;(F_{\mathrm{FS}}/F_{\mathrm{cell}}{)}_{\mathrm{wt}}}$$ (Eq 1)

$${\left(F_{cell}\right)}_{norm}=\frac{F_{\mathrm{cell}}}{\mathrm{median}\;(F_{\mathrm{cell}}{)}_{\mathrm{wt}}}$$ (Eq 2)

### Transmission electron microscopy

Samples were collected from DSM cultures at hour 4 of sporulation and processed from thin sectioning transmission electron microscopy essentially as described [61]. Samples were viewed on a Hitachi H-7650 microscope equipped with an AMT digital camera and operated at 100 keV.

### Dansylcadaverine labeling assays

Tgl (WT and mutant forms) and C30 were introduced in a pET derivative (Novagen) fused to a C-terminal His_6_-tag and protein over-production was achieved by auto-induction [15]. The different proteins were purified in 1 ml Ni^2+^-NTA Agarose columns (for details see the Supporting Material). Enzyme activity assays were conducted at 37ºC or 50ºC in Tris-HCl 0.1 M, pH 8.0, as described before [14]. The following concentrations were used: BSA (New England Biolabs), 60 μM; dansylcadaverine (Fluka), 0.5 mM; and Tgl^wt/mut^ (16 μM). BSA/Tgl labeling by dansylcadaverine was detected after SDS-PAGE resolution using a UV transilluminator (Chemidoc XRS, Biorad) and quantified using *ImageJ 1*.*37v* [62]. The values obtained for each time point were normalized by the fluorescence detected for the wild type enzyme after 120 min of incubation. All Tgl forms were purified and assayed at least three times independently.

### C30 cross-linking assays

Tgl (wild type or mutant forms, at the indicated concentrations) was incubated with C30 (at the indicated concentrations), at 37° C, in Tris-HCl 0.1 M, pH 8.0. Samples were taken at different times and resolved in 10% SDS-PAGE gels, which were subsequently stained with Coomassie brilliant blue. For graphical representation of the data, the stained SDS-PAGE gels were scanned and the amount of C30 or Tgl at each time point was quantified using *ImageJ 1*.*37v* [62]. The values obtained for each time point were normalized according to the following equation (where t represents the different time points, in minutes, in a cross-linking reaction):

$${\left(\mathrm{Protein}\;\mathrm{cross}‑\mathrm{linking}\right)}_{\mathrm{norm}}=(1‑\frac{\mathrm{protein}\;\mathrm{at}\;\mathrm{time}\;t_{t}}{\mathrm{protein}\;\mathrm{at}\;\mathrm{time}\;t_{0}})*100$$

### Small-angle X-ray scattering

Synchrotron SEC-SAXS data were collected on the BM29 ESRF beamline (Grenoble, France) using an in-line HPLC system and used to generate an average low-resolution shape representation of C30 oligomer in solution. Please see S1 Text for details.

## Supporting information

S1 Fig Accumulation of Tgl-CFP in the absence of SafA and localization of Tgl variants. **A**: samples were collected at the indicated times during sporulation, from cultures of strains expressing *tgl-cfp* in the WT background and in a congenic Δ*safA* mutant. Note that these strains carry a deletion of the *tgl* gene and express *tgl-cfp* from the non-essential *thrC* locus. A Δ*tgl* deletion strain is also included for control of the antibody specificity. Whole cell extracts were prepared, the proteins in the extracts resolved by SDS-PAGE and the gels subject to immunoblot analysis with an anti-Tgl antibody. **B**: spores from the WT (first lane) and a *tgl*::*sp* insertional mutant (second lane), along with spores of *tgl*::*sp* strains producing either Tgl^WT^ or forms of the enzyme with the indicated single Ala substitutions from the *amyE* locus were density gradient purified. The coat proteins were extracted by treatment with SDS and DTT, resolved by SDS-PAGE and the gels stained with Coomassie (top) or subject to immunoblotting with an anti-Tgl antibody (bottom). **C**: cells were collected from sporulating cultures of the indicated strains 8 hours after the induction of sporulation by resuspension, and whole cell extracts prepared. The extracts were fractionated into a mother cell and a forespore fraction, and proteins (30 μg for the mother cell fraction and 10 μg for the forespore fraction) were resolved by SDS-PAGE and subject to immunoblotting with an anti-Tgl antibody. The blots were re-probed with an anti-CotJC antibody, as a loading control. In **B** and **C**: the residues are color coded according to the position/function in the enzyme: catalytic residues, green; residues on the front (Q) side, blue; residues on the back (K) side, red. The position of relevant proteins is indicated by arrows. The asterisks show the position of possible Tgl processing products. Molecular weight markers, in kDa, are shown on the left side of all panels.
(TIFF)

S2 Fig Distribution of mutant forms of Tgl in mature spores. **A**: spores from the WT (first lane) and a *tgl*::*sp* insertional mutant (last lane), along with spores of *tgl*::*sp* strains producing either Tgl^WT^ or forms of the enzyme with the indicated single Ala substitutions from the *amyE* locus were density gradient purified. The coat proteins were extracted by treatment with SDS and DTT, resolved by SDS-PAGE and the gels stained with Coomassie (top) or subject to immunoblotting with an anti-Tgl antibody (bottom). The position of the Tgl substrates is indicated by black arrows. **B**: purified spores were decoated by boiling in a buffer containing SDS and reducing agents to produce a coat fraction. The decoated spores were incubated with lysozyme and then re-extracted, to produce a fraction called “cortex fraction” [35]. **C** and **D**: proteins in the coat (C) and cortex (D) fractions were resolved by SDS-PAGE and the gels subject to immunoblot analysis with an anti-Tgl (top panels) or an anti-CotA antibody (bottom panels). The position of Tgl and CotA is indicated by black arrows. Red arrows indicate the position of species, *a*, *b* and *c*, referred to in the supporting text. Black asterisks indicate bands that cross-react with the anti-Tgl antibody, and red asterisks possible degradation products. Possible multimeric forms of Tgl are indicated by red parenthesis. In **A** through **D**: the residues are color coded according to the position/function in the enzyme: catalytic residues, green; residues on the front (Q) side, blue; residues on the back (K) side, red. The position of molecular weight markers, in kDa, are shown on the left side of all panels.
(TIFF)

S3 Fig **Cross-linking of C30 (A) and BSA (B) by Tgl**^**WT**^. Reaction mixes containing the indicated proteins were incubated at 50ºC and samples withdrawn at the indicated times (in min) for SDS-PAGE analysis. The gels were stained with Coomassie. The blue arrow shows the position of C30, the red arrow the position of Tgl, and the grey arrow the position of BSA. The asterisk in A shows the position of a possible C30 dimer. The red parenthesis shows the position of cross-linked forms of C30 and BSA. The black arrow shows the resolving/stacking gel interface. The position of molecular weight markers (in kDa) is shown on the left side of the panels.
(TIFF)

S4 Fig Localization of YaaH-YFP in the absence of SafA^FL^. **A**: The localization of Tgl-CFP (WT or with the Y171A substitution) and YaaH-YFP was examined in a strain unable to produce full-length SafA (Δ*safA*^FL^) but producing C30-YFP from the *thrC* locus. The strains also bear a *tgl*::*sp* insertional allele and express *tgl-cfp* from the *amyE* locus. Sporulation was induced by resuspension, samples collected 8 hours thereafter, and the localization of the CFP or YFP fusions monitored by phase contrast and fluorescence microscopy. White arrowheads indicate the position of the fluorescence signal from Tgl-CFP or YaaH-YFP in cells in which a forespore could be detected (red color is used for the phase contrast images). Scale bar, 1 μm. **B.** The graph shows the percentage of sporangia of phase dark spores that showed YaaH-YFP localized as a dot of fluorescence in the MCP forespore pole (class a) and Tgl-CFP with a similar pattern (class b). Sporangia were also scored for the co-localization of YaaH-YFP and Tgl-CFP at the MCP pole of the forespore (class c). The graphs display the standard deviation for each class. A minimum of 200 cells were scored for each strain in each of two independent experiments. A diagram below the graph shows the localization patterns scored.
(TIFF)

S5 Fig Cross-linking of C30 by Tgl^WT^. **A**: Tgl, at the indicated concentrations was mixed with C30 (at 25 μM) and samples taken from the reaction mixture at the indicated times, mixed with loading dye and resolved by SDS-PAGE. **B**: as in A, except that Tgl (at 8 μM) was incubated with varying concentrations of C30, as indicated. **C**: Tgl (at 4 μM) was incubated alone and C30 (to a final concentration of 25 μM) added 20 min after. In A-C, samples were collected from the reaction mixture at the indicated times, the proteins resolved by SDS-PAGE and the level of C30 (A and B) or Tgl (C) quantified and normalized for the initial. In A-C, the gels are representative of three experiments, with different preparations of Tgl and C30. The position of molecular weight marker (in kDa) is shown on the left side of the panels. The arrows show the position of Tgl, C30, and a C30 dimer, (C30)_2_. The parenthesis shows the position of higher molecular weight forms of C30.
(TIFF)

S6 Fig C30 behaves as a hexamer in solution. **A**: C30-His_6_ was overproduced In *E*. *coli E*. *coli* BL21(DE3) from a T7_*lac*_ promoter, following an auto-induction regime and purified by Ni^2+^-NTA affinity NTA chromatography. Following purification, a sample was resolved by SDS-PAGE. The black arrow shows the position of C30 and the red arrow the position of a possible dimer. The position of molecular weight markers (in kDa) is shown on the left side of the panel. **B**: C30-His_6_ was applied to a size exclusion at a concentration of 3.5 mg/ml. C30-His_6_ eluted as a single peak corresponding to a mass of 151.14±7.5 kDa; the predicted size of C30 is 25.9 kDa and therefore this species most likely corresponds to a hexamer, (C30)_6_. The elution position of γ-globulin and a BSA dimer is shown. **C**: Comparative SAXS of the (C30)_6_ oligomer and a globular 26 kDa protein. Double-logarithmic representations of the SAXS patterns of C30 (blue circles) and Chymotrypsinogen A (purple open-circles), next to their respective *ab initio* shape representations; the superimposition is also shown (top right), highlighting the size/shape differences between the C30 SEC-purified oligomeric-state and monomeric Chymotrypsinogen A. Chymotrypsinogen A is a monomeric globular protein with a molecular mass similar to a C30 monomer (25.9 kDa). The monomeric globular protein is to scale with the C30 oligomer, and a 50 Å length scale is shown next to the molecular envelopes. The C30 oligomer in solution, with an estimated mass of 6 protomers, can accommodate six molecules of Chymotrypsinogen A. The experimental set for Chymotrypsinogen A was obtained from the curated repository for scattering data SASDB (www.sasbdb.org; entry code SASDAA8). **D:** Comparison between the *P(r)* versus *r* profiles determined from the SAXS data of C30 (red) and Chymotrypsinogen A (cyan). The derived *R*_*g*_ and *D*_*max*_ values are displayed in dashed lines.
(TIFF)

S1 Text Supporting information text. (DOCX)

S1 Table Bacterial strains used in this study. (DOCX)

S2 Table List of plasmids used in this study. (DOCX)

S3 Table Oligonucleotide primers used in this study. (DOCX)
