# Supplementary figures and images for: Temporal and spatial regulation of protein cross-linking by the pre-assembled substrates of a Bacillus subtilis spore coat transglutaminase
Source: PLoS Genet. 2019 Apr 8;15(4):e1007912. doi: 10.1371/journal.pgen.1007912 (PMC6490927; doi:10.1371/journal.pgen.1007912)

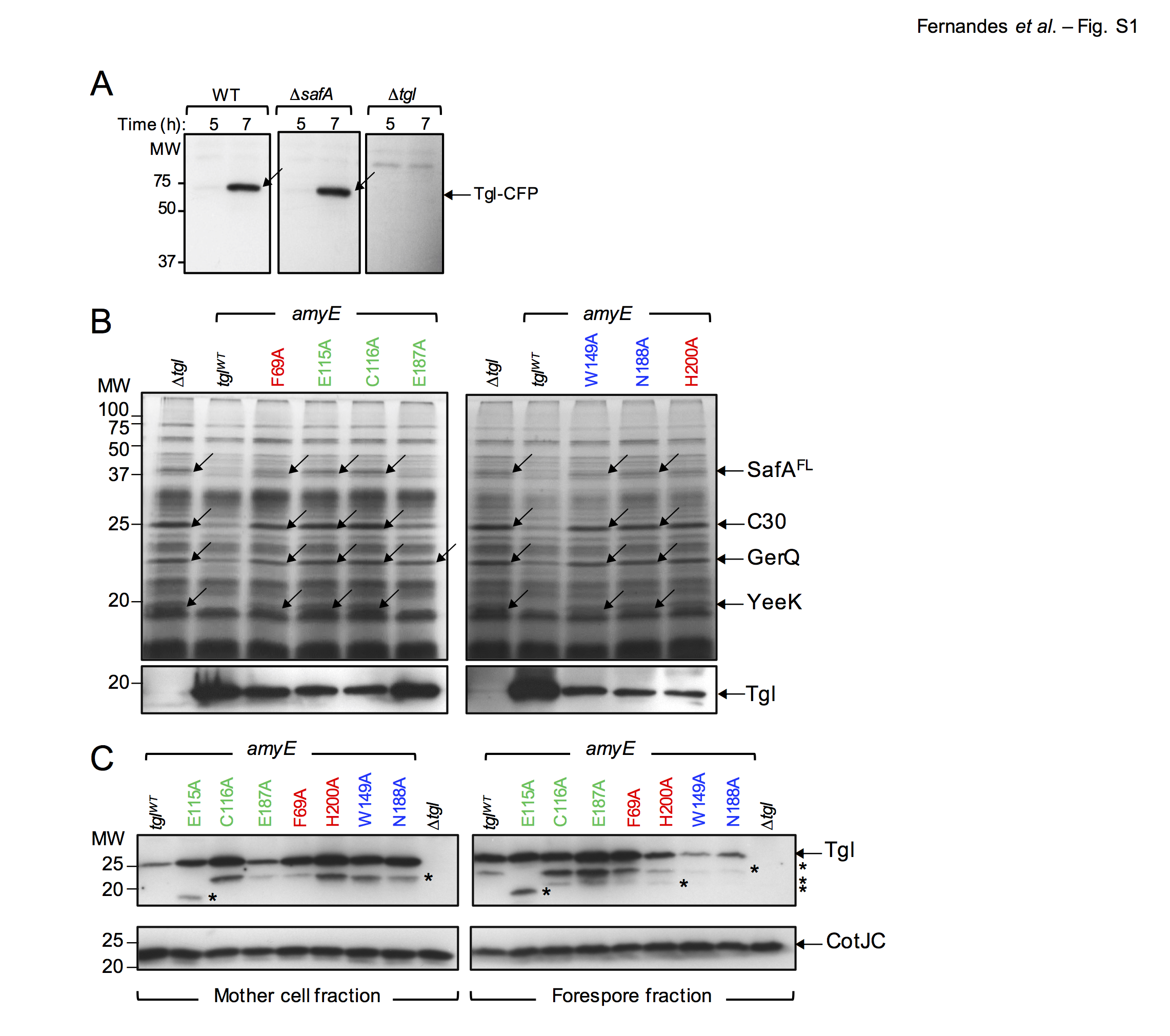

Supplement: S1 Fig — A: samples were collected at the indicated times during sporulation, from cultures of strains expressing tgl-cfp in the WT background and in a congenic ΔsafA mutant. Note that these strains carry a deletion of the tgl gene and express tgl-cfp from the non-essential thrC locus. A Δtgl deletion strain is also included for control of the antibody specificity. Whole cell extracts were prepared, the proteins in the extracts resolved by SDS-PAGE and the gels subject to immunoblot analysis with an anti-Tgl antibody. B: spores from the WT (first lane) and a tgl::sp insertional mutant (second lane), along with spores of tgl::sp strains producing either TglWT or forms of the enzyme with the indicated single Ala substitutions from the amyE locus were density gradient purified. The coat proteins were extracted by treatment with SDS and DTT, resolved by SDS-PAGE and the gels stained with Coomassie (top) or subject to immunoblotting with an anti-Tgl antibody (bottom). C: cells were collected from sporulating cultures of the indicated strains 8 hours after the induction of sporulation by resuspension, and whole cell extracts prepared. The extracts were fractionated into a mother cell and a forespore fraction, and proteins (30 μg for the mother cell fraction and 10 μg for the forespore fraction) were resolved by SDS-PAGE and subject to immunoblotting with an anti-Tgl antibody. The blots were re-probed with an anti-CotJC antibody, as a loading control. In B and C: the residues are color coded according to the position/function in the enzyme: catalytic residues, green; residues on the front (Q) side, blue; residues on the back (K) side, red. The position of relevant proteins is indicated by arrows. The asterisks show the position of possible Tgl processing products. Molecular weight markers, in kDa, are shown on the left side of all panels. (TIFF) [file pgen.1007912.s001.tiff]

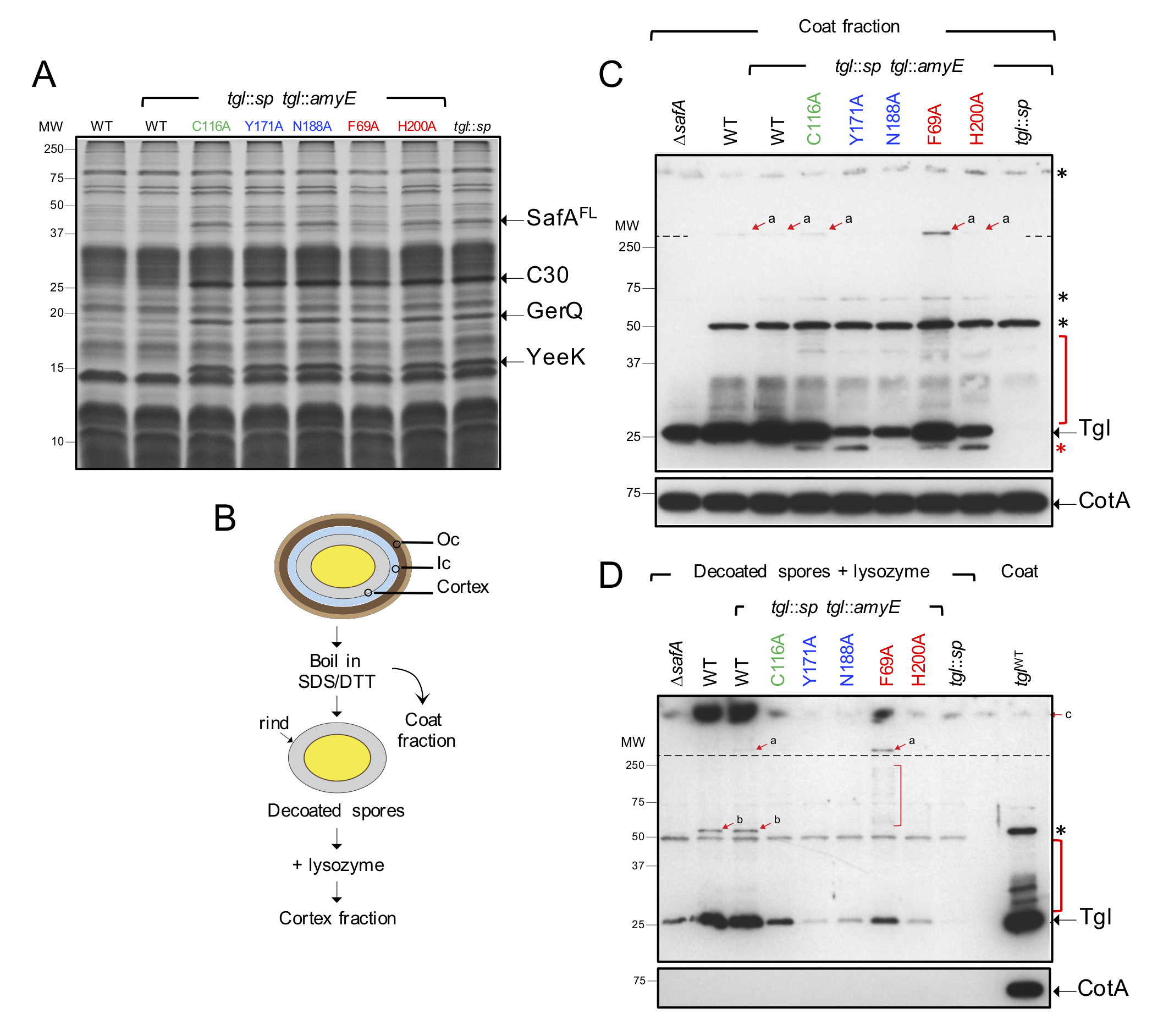

Supplement: S2 Fig — A: spores from the WT (first lane) and a tgl::sp insertional mutant (last lane), along with spores of tgl::sp strains producing either TglWT or forms of the enzyme with the indicated single Ala substitutions from the amyE locus were density gradient purified. The coat proteins were extracted by treatment with SDS and DTT, resolved by SDS-PAGE and the gels stained with Coomassie (top) or subject to immunoblotting with an anti-Tgl antibody (bottom). The position of the Tgl substrates is indicated by black arrows. B: purified spores were decoated by boiling in a buffer containing SDS and reducing agents to produce a coat fraction. The decoated spores were incubated with lysozyme and then re-extracted, to produce a fraction called “cortex fraction” [35]. C and D: proteins in the coat (C) and cortex (D) fractions were resolved by SDS-PAGE and the gels subject to immunoblot analysis with an anti-Tgl (top panels) or an anti-CotA antibody (bottom panels). The position of Tgl and CotA is indicated by black arrows. Red arrows indicate the position of species, a, b and c, referred to in the supporting text. Black asterisks indicate bands that cross-react with the anti-Tgl antibody, and red asterisks possible degradation products. Possible multimeric forms of Tgl are indicated by red parenthesis. In A through D: the residues are color coded according to the position/function in the enzyme: catalytic residues, green; residues on the front (Q) side, blue; residues on the back (K) side, red. The position of molecular weight markers, in kDa, are shown on the left side of all panels. (TIFF) [file pgen.1007912.s002.tiff]

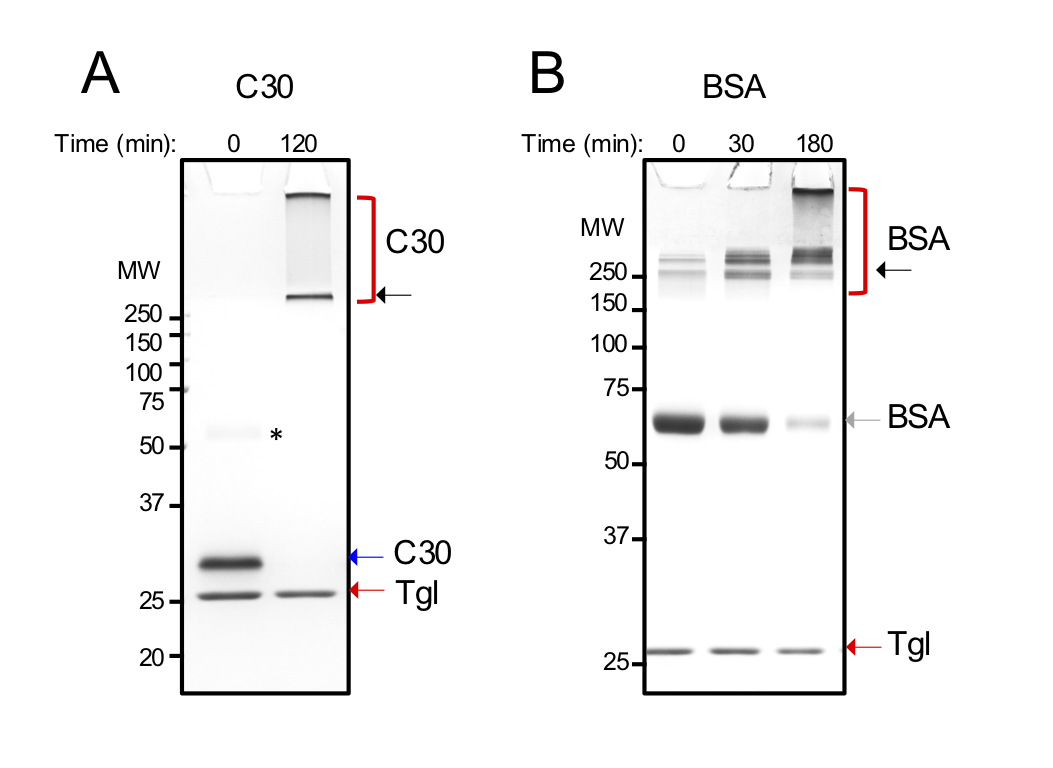

Supplement: S3 Fig — Cross-linking of C30 (A) and BSA (B) by TglWT. Reaction mixes containing the indicated proteins were incubated at 50ºC and samples withdrawn at the indicated times (in min) for SDS-PAGE analysis. The gels were stained with Coomassie. The blue arrow shows the position of C30, the red arrow the position of Tgl, and the grey arrow the position of BSA. The asterisk in A shows the position of a possible C30 dimer. The red parenthesis shows the position of cross-linked forms of C30 and BSA. The black arrow shows the resolving/stacking gel interface. The position of molecular weight markers (in kDa) is shown on the left side of the panels. (TIFF) [file pgen.1007912.s003.tiff]

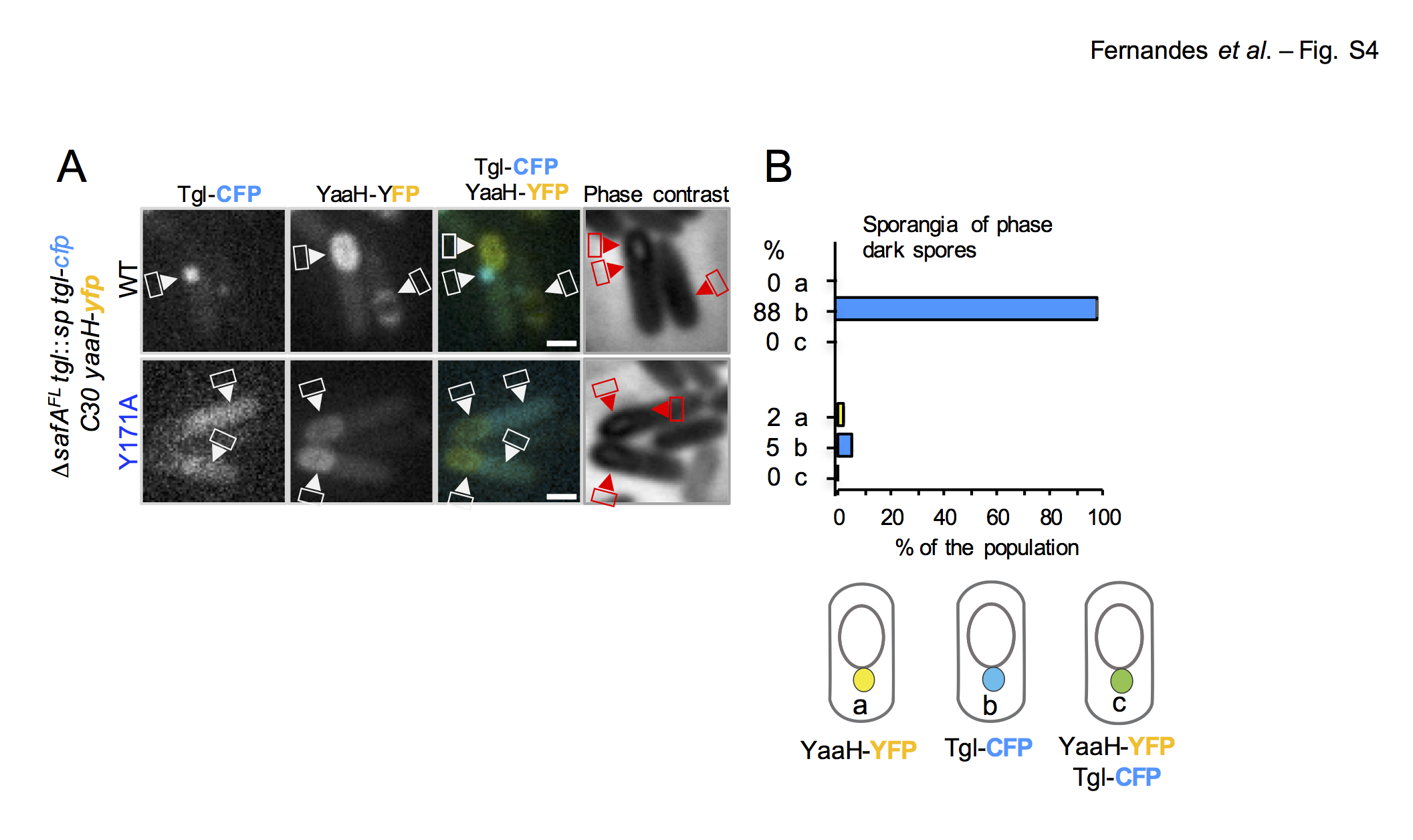

Supplement: S4 Fig — A: The localization of Tgl-CFP (WT or with the Y171A substitution) and YaaH-YFP was examined in a strain unable to produce full-length SafA (ΔsafAFL) but producing C30-YFP from the thrC locus. The strains also bear a tgl::sp insertional allele and express tgl-cfp from the amyE locus. Sporulation was induced by resuspension, samples collected 8 hours thereafter, and the localization of the CFP or YFP fusions monitored by phase contrast and fluorescence microscopy. White arrowheads indicate the position of the fluorescence signal from Tgl-CFP or YaaH-YFP in cells in which a forespore could be detected (red color is used for the phase contrast images). Scale bar, 1 μm. B. The graph shows the percentage of sporangia of phase dark spores that showed YaaH-YFP localized as a dot of fluorescence in the MCP forespore pole (class a) and Tgl-CFP with a similar pattern (class b). Sporangia were also scored for the co-localization of YaaH-YFP and Tgl-CFP at the MCP pole of the forespore (class c). The graphs display the standard deviation for each class. A minimum of 200 cells were scored for each strain in each of two independent experiments. A diagram below the graph shows the localization patterns scored. (TIFF) [file pgen.1007912.s004.tiff]

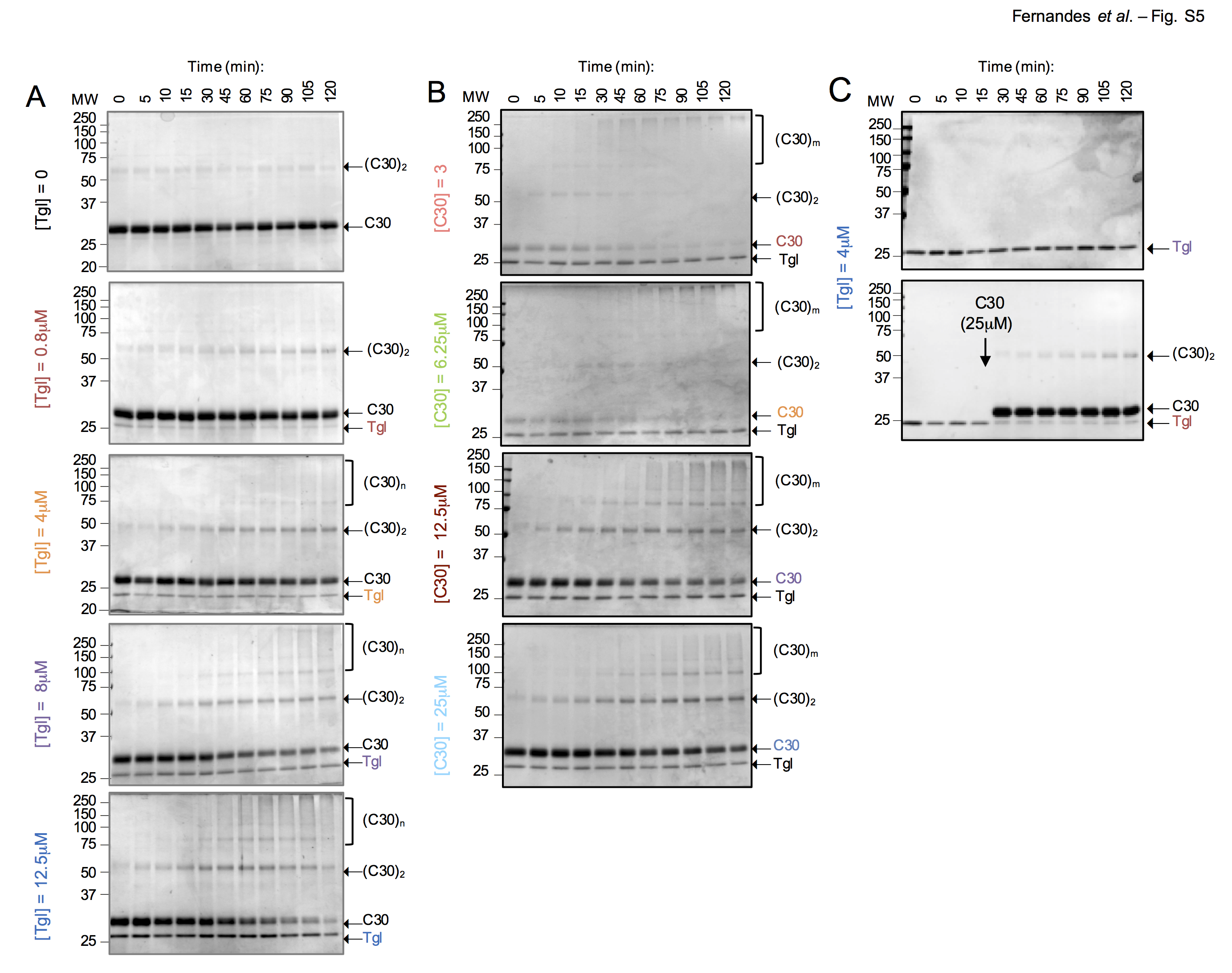

Supplement: S5 Fig — A: Tgl, at the indicated concentrations was mixed with C30 (at 25 μM) and samples taken from the reaction mixture at the indicated times, mixed with loading dye and resolved by SDS-PAGE. B: as in A, except that Tgl (at 8 μM) was incubated with varying concentrations of C30, as indicated. C: Tgl (at 4 μM) was incubated alone and C30 (to a final concentration of 25 μM) added 20 min after. In A-C, samples were collected from the reaction mixture at the indicated times, the proteins resolved by SDS-PAGE and the level of C30 (A and B) or Tgl (C) quantified and normalized for the initial. In A-C, the gels are representative of three experiments, with different preparations of Tgl and C30. The position of molecular weight marker (in kDa) is shown on the left side of the panels. The arrows show the position of Tgl, C30, and a C30 dimer, (C30)2. The parenthesis shows the position of higher molecular weight forms of C30. (TIFF) [file pgen.1007912.s005.tiff]

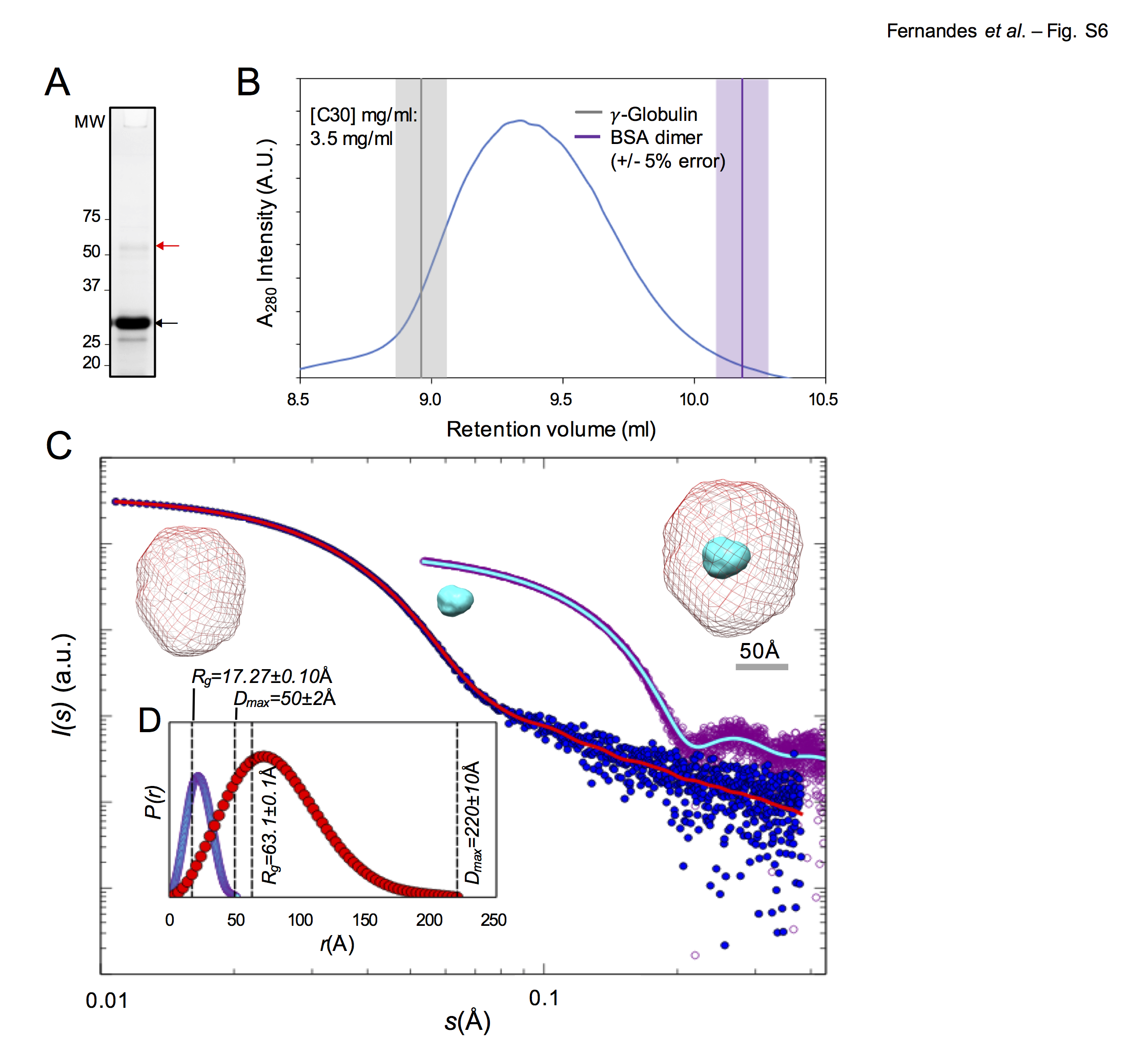

Supplement: S6 Fig — A: C30-His6 was overproduced In E. coli E. coli BL21(DE3) from a T7lac promoter, following an auto-induction regime and purified by Ni2+-NTA affinity NTA chromatography. Following purification, a sample was resolved by SDS-PAGE. The black arrow shows the position of C30 and the red arrow the position of a possible dimer. The position of molecular weight markers (in kDa) is shown on the left side of the panel. B: C30-His6 was applied to a size exclusion at a concentration of 3.5 mg/ml. C30-His6 eluted as a single peak corresponding to a mass of 151.14±7.5 kDa; the predicted size of C30 is 25.9 kDa and therefore this species most likely corresponds to a hexamer, (C30)6. The elution position of γ-globulin and a BSA dimer is shown. C: Comparative SAXS of the (C30)6 oligomer and a globular 26 kDa protein. Double-logarithmic representations of the SAXS patterns of C30 (blue circles) and Chymotrypsinogen A (purple open-circles), next to their respective ab initio shape representations; the superimposition is also shown (top right), highlighting the size/shape differences between the C30 SEC-purified oligomeric-state and monomeric Chymotrypsinogen A. Chymotrypsinogen A is a monomeric globular protein with a molecular mass similar to a C30 monomer (25.9 kDa). The monomeric globular protein is to scale with the C30 oligomer, and a 50 Å length scale is shown next to the molecular envelopes. The C30 oligomer in solution, with an estimated mass of 6 protomers, can accommodate six molecules of Chymotrypsinogen A. The experimental set for Chymotrypsinogen A was obtained from the curated repository for scattering data SASDB (www.sasbdb.org; entry code SASDAA8). D: Comparison between the P(r) versus r profiles determined from the SAXS data of C30 (red) and Chymotrypsinogen A (cyan). The derived Rg and Dmax values are displayed in dashed lines. (TIFF) [file pgen.1007912.s006.tiff]
