# Supplementary material for: Temporal and spatial regulation of protein cross-linking by the pre-assembled substrates of a Bacillus subtilis spore coat transglutaminase
Source: PLoS Genet. 2019 Apr 8;15(4):e1007912. doi: 10.1371/journal.pgen.1007912 (PMC6490927; doi:10.1371/journal.pgen.1007912)
Supplement: S1 Text — (DOCX) [file pgen.1007912.s007.docx]

**S1 Text**

**Supporting Information**

**Temporal and spatial regulation of protein cross-linking by the pre-assembled substrates of a *Bacillus subtilis* spore coat transglutaminase**

Catarina G. Fernandes^1#^, Diogo Martins^1$^, Guillem Hernandez^1$^, Ana L. Sousa^2^, Carolina Freitas^1, ##^, Erin M. Tranfield^2^, Tiago N. Cordeiro^1^, Mónica Serrano^1^, Charles. P. Moran Jr.^3*^, and Adriano O. Henriques^1,^*

^1^Microbial Development Group, Instituto de Tecnologia Química e Biológica António Xavier, Universidade Nova de Lisboa, ITQB-UNL, 2780-157 Oeiras, Portugal.

^2^Electron Microscopy Facility, Instituto Gulbenkian de Ciência, 2780-156 Oeiras, Portugal

^3^Emory University School of Medicine, Atlanta GA 30322, USA

^#^Present address: Hovione FarmaCiencia SA, Estrada do Paço do Lumiar, 1649-038 Lisboa, Portugal.

^##^Present address: Department of Ecophysiology, Max-Planck Institute for Terrestrial Microbiology, Karl-von-Frisch-Str. 10, D-35043 Marburg, Germany.

*Corresponding authors: Phone: +351 21 4469521 or +14047275969; Fax: +351 21 441 or +1 4047273659; *Email*: [aoh@itqb.unl.pt](mailto:aoh@itqb.unl.pt) or cmoran@emory.edu

^$^Equivalent contributions.

**Supporting Material and Methods**

**Bacterial strains, media and general methods*.*** *Escherichia coli* DH5α was used for plasmid constructions. The strains of *Bacillus subtilis* used in this study were derived from the wild type MB24 strain. Luria-Bertani medium was used for routine growth of *E. coli* and *B. subtilis* strains. For construction of plasmids, NZYDNAChange polymerase (NZYTech) was used for the amplification of fragments, while Phusion high-fidelity DNA polymerase (Finnzymes) was used when whole plasmids where amplified. Restriction enzymes from Fermentas were used. With the exception of plasmids created by sub-cloning strategies, all plasmids constructed were sequenced. For the construction of *B. subtilis* strains, insertion of genes at the *amyE* locus were confirmed by loss of extracellular amidase activity and by PCR if necessary; insertions at other loci were confirmed by PCR, except when otherwise stated or when a resistance cassette gene was substituted [[63](#_ENREF_63)]. All strains, plasmids and primers used in this study are listed in S1, S2 and S3, Tables respectively.

**Spore fractionation and fractionation of sporulating cells**. Spores were purified from 24 hours cultures of the relevant strains in DSM, using a two-step gradient of Gastrografin (Bayer Schering Pharma) [[35](#_ENREF_35)]. The preparation of coat protein extracts and the fractionation of spores were described recently [[35](#_ENREF_35)]. For the fractionation of sporulating cells, cultures of the various strains were grown at 37°C in DSM without antibiotics and samples (15 ml) taken 6 hours after the initiation of sporulation. The samples were used to prepare a whole cell lysate, as well as a mother cell and a forespore fraction as described [[35](#_ENREF_35)].

**Overproduction and purification of wild type and mutant forms of Tgl*.*** Point mutations were introduced in the *tgl* gene present in pLOM4 [[31](#_ENREF_31)], a pET30a(+) (Novagen) derivative where *tgl* is fused to a C-terminal His_6_-tag, and its expression is under the control of a T7*lac* promoter. The following set of primers were used to introduce alanine substitutions (F69A, W149A, Y171A, W184A, R185A and N188A) in *tgl*: Tgl F69Adir and Tgl F69Arev, Tgl W149dir and Tgl W149rev, Tgl Y171Adir and Tgl Y171Arev, Tgl W184Adir and Tgl W184Arev, Tgl R185Adir and Tgl R185Arev, and Tgl N188Adir and Tgl N188Arev, creating plasmids pCF19, pCF8, pCF25, pCF20, pCF21 and pCF9, respectively (S2Table). These plasmids were introduced in *E. coli* BL21(DE3) (New England Biolabs) for the production of Tgl with the different single Ala substitutions. The only exception was pCF19 which was introduced in *E. coli* C43 [[35](#_ENREF_35), [64](#_ENREF_64)], as production of Tgl^F69A^ in *E. coli* BL21(DE3) caused extensive cell lysis. All forms of Tgl were overproduced using an auto-induction regime, purified using 1 ml columns packed with Ni^2+^-NTA Agarose resin (Qiagen), and dialyzed against 0.1 M Tris-HCl (pH 8.0) ([[14](#_ENREF_14)]; see below).

**Introduction of *tgl_mut_-cfp* at the *thrC* locus.** pCF89 (which was designed to transfer a *tgl*-*cfp* fusion to the non-essential *thrC* locus) was amplified with primers tgl+288D and tgl+453R, eliminating base pairs 313 to 594 of the coding region of *tgl*, yielding pCF100. *tgl*^E115A^ and *tgl*^E187A^ were obtained by the digestion of pCF43, or pCF12, respectively, with *Sfo*I and *EcoR*I, and the fragmentes were inserted between the same sites of pCF100, creating plasmids pCF104 and, pCF105. Primers Tgl F69Adir and tgl+735D were used to amplify *tgl*^C116A^, *tgl*^H200A^, *tgl*^W149A^, *tgl*^N188A^, *tgl*^W184A^, *tgl*^R185A^ and *tgl*^Y171A^ from plasmids pCF1, pCF2, pCF8, pCF9, pCF20, pCF21, and pCF25, respectively. The fragments were digested with *Sfo*I and *EcoR*I and cloned between the same sites of pCF100, creating plamids pCF114, pCF115, pCF116, pCF107, pCF121, pCF122, and pCF123. The cloning of the different fragments in pCF100 restores the *tgl* gene. Finally, primers Tgl F69Adir and Tgl F69Arev were used to amplify pCF89 which exchanges codon 69 of *tgl* from Phe to Ala, yielding pCF102.

**Overproduction and purification of C30*.*** The coding region of C30 was amplified from genomic DNA of MB24, with primers safA+477D and safA+1176R. The resulting fragment was digested with *Nco*I and *Xho*I and introduced between the same sites of pET28a(+) (Novagen). This produced pCF68, containing *C30* fused to a C-terminal His_6_-tag and whose expression is under the control of a T7*lac* promoter, induced by IPTG or lactose. For production of C30, pCF68 was introduced in *E. coli* BL21(DE3) and the strain was grown at 37°C, for ~18 hr, in a modified auto-induction medium supplemented with 100 μg/ml of kanamycin (as described for Tgl; [[14](#_ENREF_14), [15](#_ENREF_15)]). The cells were harvested by centrifugation (5 min at 15,300 x g, 4° C) and resuspended in 1/10 of the culture volume with lysis buffer (50 mM NaH_2_PO_4_, 0.5 M NaCl, 10 mM Imidazole, pH 8.0). Cells were disrupted by 2 passages in a French Press cell (19,000 lb/in^2^). Unbroken cells and cellular debris were cleared by centrifugation (30 min at 27,200 x g, 4° C). The clear lysate was applied to 1 ml columns packed with Ni^2+^-NTA Agarose resin (Qiagen) previously equilibrated with lysis buffer. The column was washed with 10 ml of three different buffers: wash 1, lysis buffer containing 10% glycerol; wash 2, lysis buffer with 60 mM imidazole; and wash 3, lysis buffer with 100 mM imidazole. Finally, C30 was eluted with lysis buffer containing 250 mM imidazole. The purified fractions of C30 where dialyzed overnight against 0.1 M Tris-HCl, 2 mM DTT, pH 8.0 (using a 10 kDa cutoff *SnakeSkin* membrane, from Pierce) and maintained at -20ºC until the time of use.

**Production of anti-SafA antibodies**. We first introduced pOZ33, carrying *safA* fused to an N-terminal His_6_ tag in pET30-a(+) [[27](#_ENREF_27)] into *E. coli* BL21(DE3) (Novagen). The cells were grown in LB to midlog phase (OD_600_ ~0.6) indiced with 1 mM IPTG for 3 hours and proteins purified by Ni^2+^-NTA affinity chromatography. Induction resulted in the accumulation of the three forms of the protein: SafA^FL^, C30 and N21, but only SafA^FL^ and N21 carry the His_6_. Following elution, the proteins were resolved by SDS-PAGE, the N21 band excised and sent to Eurogentec (Belgium) for the production of a rabbit polyclonal antibody.

**Immunoblot analysis**. Immunoblot analysis was conducted using the SuperSignal West Pico Chemiluminiscent Substrate kit (Thermo Scientific) and following the manufacturer´s instructions, using low fat powder milk and Tween-20 (0.001%) as the blocking agents [[35](#_ENREF_35)]. The antibodies were used at the following dilutions [[35](#_ENREF_35)]: anti-Tgl, 1:15000; anti-CotJC and anti-N21, 1:1000; anti-SafA, 1:25000. Secondary peroxidase-conjugated antibodies were used at a concentration of 1:100000.

**Size exclusion chromatography**. A Superdex200 (10/300 GL) 24mL column (GE Healthcare) and AKTA Purifier system were used to perform size exclusion chromatography (SEC) at 4ºC. The column was equilibrated with SEC buffer (100mM Tris·HCl pH=8, 150mM NaCl and 2mM DTT) and 450µL of C30 protein were injected under a flow of 0.5mL/min for 25 mL collecting 0.5mL fractions. Under the same conditions, we performed a column calibration using BSA (monomer 66kDa, dimer 132kDa), Lysozyme (14.4 kDa) and Gel Filtration Standard (Bio-Rad) (Thyroglobulin (bovine) 670kDa; γ-globulin (bovine) 158kDa; Ovalbumin (chicken) 44kDa; Myoglobin (horse) 17kDa; Vitamin B12 1.35kDa). To assess the accuracy of the calibration, we back-calculated the mass of newly purified BSA (monomer and dimer) and Lysozyme, giving less than 10% error. C30 eluted as a single peak, with an estimated mass of 151.4 ± 7.5 kDa, possibly corresponding to a hexamer (the predicted size of the monomer is 25.9 kDa and the expected mass of the hexamer would thus be of 155.4 kDa); this species, herein termed (C30)_6_ was used for the SAXS experiments described below.

**Small-angle X-ray scattering.** Synchrotron SEC-SAXS data were collected on the BM29 ESRF beamline (Grenoble, France) using an in-line HPLC system (Shimadzu). 45 μL sample with 10.0 mg/ml of C30 was injected into a 2.4 ml Superdex200 3.2/300 (GE Healthcare) size exclusion column at a flow rate of 0.10 mL min^−1^. The SEC mobile phase consisted of 100 mM Tris at pH 8, 150 mM NaCl, 2mM of DTT at 20ºC (see above). One-second frames were acquired using a Pilatus 1M pixel detector (DECTRIS) at a sample-detector distance of 2.8 m and a wavelength of λ = 0.99 Å [[47](#_ENREF_47)], covering a momentum of transfer range of 0.0038 < *s* < 0.49 Å^-1^. The scattering intensities from (C30)_6_ elution peak region (125-frames) were integrated, the buffer subtracted and averaged using the ScÅtter software to produce the averaged SEC-SAXS profile. From this profile, the pair-wise distance distribution function, *P(r)*, was obtained by indirect Fourier Transform with GNOM using a momentum transfer range of 0.010 < *s* <0.38 Å^-1^. The *R_g_* values were estimated by applying the Guinier approximation in the range *s* < 1.3/*R_g_*. Low-resolution *ab initio* molecular envelopes were generated with the program DAMMIF [[65](#_ENREF_65)] which uses simulated annealing to identify arrangements of densely packed dummy atoms that fit the SAXS data. We built twenty independent *ab initio* reconstructions of C30 oligomer by the “fast” annealing mode with default parameters to avoid shape bias. Models were refined with DAMNIN [[65](#_ENREF_65)], averaged, aligned, and compared using DAMAVER [[66](#_ENREF_66)]. Comparison of the models was performed using the normalized spatial discrepancy, NSD. Generally, an average NSD 1 indicates that the models are similar, while the NSD significantly exceeding one suggests large variations between the individual reconstructions. To assess the quality of the model fits we compute point-by-point residual deviation and the reduced χ^2^ test.

**Supporting Results and Discussion**

**Assembly and activity of Tgl^WT^ and mutant forms of the enzyme in vivo*.***

Spores of a *tgl*::*sp* mutant and of a *tgl*::*sp* mutant expressing *tgl^wt^* from *amyE* (reference strain), as well as spores of strains expressing the various mutant alleles of *tgl* from *amyE*, were purified and the coat proteins extracted and resolved by SDS-PAGE. The coat protein profile obtained for the *tgl*::*sp* mutant shows the characteristic increased extractability of SafA^FL^, C30, GerQ and YeeK [[31](#_ENREF_31), [35](#_ENREF_35)], but expression of *tgl^wt^* from *amyE* restored the WT pattern of extractable coat proteins (S1A Fig). We then examined the pattern of proteins obtained for various mutant alleles of *tgl*, expressed from the *amyE* locus. In general, the known Tgl-dependent proteins were more extractable from spores of the strains producing forms of Tgl with single Ala substitutions of front side (W149A, N188A), back side (F69A, H200A) and catalytic residues (E115A, C1116A) (S1A Fig). One exception, was the E187A substitution, from which SafA^FL^ was not more extractable than from spores of the reference strain (*tgl::sp* with *tgl^WT^* at *amyE*) (S1A Fig; see also the following section). The increased extractability of the Tgl-dependent proteins caused by the substitution of the catalytic Cys residue, C116A, was described before [[31](#_ENREF_31)]. With the exception of of Tgl^E187A^ form (below) whose levels were higher, in all other mutants, the level of Tgl detected in the spore coat extracts by immunoblotting was reduced relative to the reference strain, consistent with impaired assembly and with the microscopy results described in the main text (S1A Fig; see also Fig. 4).

We also examined the accumulation of the various forms of Tgl forms in mother cell and forespore fractions obtained from cells collected at hour 6 of sporulation, a time at which the two cells can be efficiently separated [[31](#_ENREF_31), [35](#_ENREF_35)]. Proteins in the extracts (30 µg for the mother cell fraction and 10 µg for the forespore fraction) were resolved by SDS-PAGE and subject to immunoblotting with an anti-Tgl antibody. All forms of the enzyme showed reduced levels in the forespore fraction (and correspondingly higher levels in the mother cell fraction) compared to the reference strain (S1B Fig). Strikingly the front side substitutions W49A and N188A resulted in the lowest levels of Tgl in the forespore fraction (see the main text). Overall, these results are in agreement with the fluorescence microscopy analysis (as described in the main text) and with the proposal that residues at the front and back side of the Tgl tunnel, as well as the active site residues located in the middle of the tunnel, are important for the assembly of the enzyme, with front side (Q side) residues making the most important contribution to the assembly of Tgl.

**The partially redundant catalytic dyad discriminates among Tgl substrates.**

In coat extracts prepared form spores of the *tgl^E187A^* mutant, only GerQ shows increased extractability comparable to *tgl*::*sp* spores (S1A Fig). In contrast, SafA^FL^ or YeeK do not show increased extractability and C30 is slightly less extractable than from *tgl*::*sp* spores (S1A Fig). Tgl has a partially redundant catalytic dyad in which C116 is always essential for catalysis, but Glu187 can be non-recriprocally substituted by Glu115 [[14](#_ENREF_14)]. Glu187 serves as the primary proton acceptor for Cys116, but Glu115, in the close vicinity of Cys116, can substitute for Glu187. Accordingly, Tgl^E187A^ still showed activity in both amine incorporation and cross-linking assays [[14](#_ENREF_14)]; the reciprocal substitution, E115A, however, completely eliminated amine incorporation and cross-linking activity, because in the mutant, Glu187 interacts with equal frequency with Cys116 and another residue in close proximity (H200), decreasing the ability of Glu187 to deprotonate the catalytic Cys116 [[14](#_ENREF_14)]. We now show that E115 is also essential for the normal assembly of the four known physiological substrates of Tgl (S1A Fig). We have proposed before that in Tgl, like in papain, which also functions through a dual catalytic dyad, catalysis could take place from distinct steric positions, and we have further suggested that perhaps different Tgl physiological substrates would be handled by one or the other of the catalytic dyads [[14](#_ENREF_14)]. The results in figure S1A suggest that Tgl^E187A^ is not able to use GerQ as a substrate *in vivo*, but can still use SafA^FL^, YeeK and, to some extent, also C30. Presumably then, SafA^FL^, YeeK and C30, albeit less efficiently, are cross-linked by Tgl using the Cys116/Glu115 catalytic dyad, at least in the context of the E187A substitution [[14](#_ENREF_14)]. The increased levels of Tgl^E187A^ relative to the other mutant forms of the enzyme may thus result from efficient interactions, resulting in assembly, with SafA^FL^, YeeK and C30.

In the main text, we suggest that the main role of the YeeK and GerQ substrates is to control the kinetics of encasement by Tgl-CFP while SafA^FL^ and C30 serve as the main recruiters fo Tgl to the surface of the developing spore (Fig. 2). That Tgl^E187A^ is extracted from the spore coat at levels nearly identical to those observed for the control strain, expressing *tgl^wt^* from *amyE* (S1A Fig.), is in agreement with the more modest role of the *gerQ* mutation in recruitment of the enzyme (Fig. 2). It also lends support to the idea that SafA^FL^ and C30 make the more important contributions to the recruitment of Tgl to the forespore.

**Distribution of Tgl^WT^ and mutant forms of the enzyme in mature spores*.***

In previous work, we have described two stages in the assembly of Tgl. First, in a manner largely but not exclusively controlled by *safA*, Tgl is recruited to the coat. Secondly, Tgl becomes associated with a “lysozyme fraction”, which most likely represents proteins that are embedded in the spore cortex and/or located at the cortex/inner coat interface; this step is completely dependent on *safA* [[35](#_ENREF_35)]. Then, Tgl cross-links SafA^FL^ and C30 in both the coat and the cortex fraction [[35](#_ENREF_35)]. Thus, we predicted that the single Ala substitutions in Tgl affecting its recruitment to the forespore also affected the distribution of the enzyme between the two spore fractions. Moreover, the substitutions of front- (Q) side residues could more markedly affect the association of Tgl with the cortex, as this localization is totally dependent on SafA. We purified spores of the WT and *tgl*::*sp* strains, along with spores of strains expressing front-side (Y171A, N188A), back-side (F69A, H200A) and catalytic mutants (C116A) from *amyE* (S2A Fig). The spores were decoated to produce a coat fraction, and then treated with lysozyme and re-extracted, to produce a “cortex fraction”, as depicted in figure S2B and described previously [[35](#_ENREF_35)]. Proteins in the two fractions were resolved by SDS-PAGE and then subject to immunoblot analysis with anti-Tgl antibodies and also with an anti-CotA antibody to assess the presence of CotA, a well characterized outer coat protein, in the coat fraction (S2C Fig) and its absence from the “cortex fraction” (S2D Fig.) [[35](#_ENREF_35)].

In the coat fraction of the WT or the reference strain (the *tgl*::*sp* mutant expressing *tgl^WT^* from *amyE*) spores, Tgl was found as two main forms: a band of about 27 KDa, consistent with the predicted size of the protein (28.3 kDa) and a species of apparent mass >250 kDa, at the resolving/stacking gel interface (S2C Fig., band *a*; the broken line marks the resolving/stacking gel interface). The 27 kDa species was detected for all the mutants, but was reduced for the front-side Y171A and N188A mutants and for the back-side mutant H200A, but not for the F69A or C116A mutants (S2C Fig.). Band *a* was not detected for the two front-side mutants tested (S2C Fig.). Its presence in the C116A mutant indicates however, that it does not result from auto-cross-linking activity. These results are in general agreement with the view that the residues at the front-side of the Tgl tunnel make a more important contribution to the assembly of the enzyme than catalytic or back-side residues. They also suggest that the front-side substitutions impair assembly of Tgl at the coat recruitment step, while other substitutions may affect more pronouncedly a subsequent step.

In the “cortex fraction” of spores of the WT or the reference strain, Tgl is found as a main species of 27 kDa, and as three additional species: *a* migrates at the resolving/stacking gel and is probably equivalent to the species seen at this region of the gel in the coat fraction; *b* has an apparent mass of about 55 kDa; *c* accumulates at the origin of the gel. Species *a*, *b* and *c* were absent or greatly reduced in all the mutants, with the exception of F69A in which all of the three forms were detected (S2D Fig). The 27 kDa form was also reduced, relative to the reference strain, for all mutants; this reduction however, was more pronounced for the Y171A, N188A (front-side residues) than for C116A and F69A (a back-side residue) (S3D Fig.). This is in line with the proposal that front-side residues make a more important contribution to the assembly of Tgl, and with the idea that a key step in the assembly of Tgl is its association with a “cortex fraction” [[35](#_ENREF_35)]. We note, however, that the 27 kDa form of Tgl in the “cortex fraction” was as reduced for the H200A mutant as it was for the front-side mutants Y171A and N188A (S2D Fig). One possibility is that this residue is important for the interaction of Tgl with a substrate that is more represented in the “cortex fraction”.

Tgl is first recruited to the coat mainly (but not exclusively) by SafA and in a second stage associates with the “cortex fraction” in a manner that is absolutely dependent on *safA*; Tgl then cross-links SafA^FL^ and C30 in the two fractions, although mainly in the “cortex fraction” where both forms of SafA also seem to be more abundant [[35](#_ENREF_35)]. Note that as described before, Tgl is greatly reduced in the “cortex fraction” of Δ*safA* spores (S2D Fig) [[35](#_ENREF_35)]. The apparent greater impact of the single Ala substitutions of front-, back-side and catalytic residues on the association of Tgl with the “cortex layer” is thus in line with the greater representation of SafA^FL^ and C30 in this fraction, with the role of these two proteins as the main recruiters of Tgl, and with their essentiality in drafting Tgl to the cortex [[35](#_ENREF_35)].

**Cross-linking of C30 and BSA at 50ºC.**

Purified C30 and BSA (commercial preparation, from Sigma) were incubated with Tgl at 50ºC for 120 min in the case of C30, and for 30 and 180 min in the case of BSA. The cross-linked products were resolved by SDS-PAGE and the gels stained with Coomassie. All of the C30 protein present at the beginning of the incubation was converted into products that either migrated at the resolving/stacking gel interface, or that did not enter the stacking gel (S3A Fig.). Most of the BSA was converted, over time, into products that run at the resolving/stacking gel interface or stayed at the origin of the gel (S3B Fig.). C30 is thus more efficiently cross-linked than BSA, which showed incomplete cross-linking even upon incubation with Tgl for 180 min (as compared to 120 min for C30).

**References**

63. Steinmetz M, Richter R. Plasmids designed to alter the antibiotic resistance expressed by insertion mutations in Bacillus subtilis, through in vivo recombination. Gene. 1994;142(1):79-83. PubMed PMID: 8181761.

64. Miroux B, Walker JE. Over-production of proteins in Escherichia coli: mutant hosts that allow synthesis of some membrane proteins and globular proteins at high levels. J Mol Biol. 1996;260(3):289-98. doi: 10.1006/jmbi.1996.0399. PubMed PMID: 8757792.

65. Franke D, Svergun DI. DAMMIF, a program for rapid ab-initio shape determination in small-angle scattering. J Appl Crystallogr. 2009;42(Pt 2):342-6. doi: 10.1107/S0021889809000338. PubMed PMID: 27630371; PubMed Central PMCID: PMCPMC5023043.

66. Volkov VV, Svergun, D.I. Uniqueness of ab initio shape determination in small-angle scattering. J Appl Cryst 2003;36:860–4.
