# Supplementary material for: Temporal and spatial regulation of protein cross-linking by the pre-assembled substrates of a Bacillus subtilis spore coat transglutaminase
Source: PLoS Genet. 2019 Apr 8;15(4):e1007912. doi: 10.1371/journal.pgen.1007912 (PMC6490927; doi:10.1371/journal.pgen.1007912)
Supplement: S1 Table — (DOCX) [file pgen.1007912.s008.docx]

| **S1 Table. Bacterial strains used in this study.** | | |
| --- | --- | --- |
| Strain | Genotype and phenotype | Origin/Construction |
| ***B. subtilis*** | | |
| MB24 | *trpC2 metC3*, wild type | Laboratory Stock |
| AH2203 | *trpC2 metC3* *gerQ::cm,* Cm^R^ | [[30](#_ENREF_30)] |
| AH2255 | *trpC2 metC3* *tgl::sp,* Sp^R^ | “ |
| AH10331 | *trpC2 metC3* *tgl::sp* Δ*thrC::tgl-hl4-cfp*(-35), Sp^R^, Erm^R^ | [[34](#_ENREF_34)] |
| AH10281 | *trpC2 metC3* *yeeK::cm*, Cm^R^ | MB24 x pCF65 |
| AH10282 | *trpC2 metC3* *yeeK::cm* Δ*tgl::sp*, Sp^R^, Cm^R^ | AH2255 x AH10281 |
| AH10352 | *trpC2 metC3* *tgl::sp* Δ*safA* Δa*myE::cm*, Sp^R^, Cm^R^ | [[34](#_ENREF_34)] |
| AH10354 | *trpC2 metC3* *yeeK::tet*, Tet^R^ | AH10281 x pCm::Tc |
| AH10355 | *trpC2 metC3* *gerQ::neo*, Neo^R^ | AH2203 x pCm::Nm |
| AH10357 | *trpC2 metC3* *tgl::sp* Δ*thrc::tgl-hl4-cfp*(-35) Δ*safA* Δa*myE::cm* Sp^R^, Erm^R^, Cm^R^ | [[34](#_ENREF_34)] |
| AH10361 | *trpC2 metC3* *tgl::sp ΔthrC::cm*, Sp^R^, Cm^R^ | “ |
| AH10366 | *trpC2 metC3* *tgl::sp ΔthrC::tgl*^F69A^*-hl4-cfp*(-35), Sp^R^, Erm^R^ | AH10361 x pCF102 |
| AH10367 | *trpC2 metC3* *tgl::sp ΔthrC::tgl*^E115A^*-hl4-cfp*(-35), Sp^R^, Erm^R^ | AH10361 x pCF104 |
| AH10370 | *trpC2 metC3* *tgl::sp ΔthrC::tgl*^E187A^*-hl4-cfp*(-35), Sp^R^, Erm^R^ | AH10361 x pCF105 |
| AH10373 | *trpC2 metC3* *tgl::sp ΔthrC::tgl*^N188A^*-hl4-cfp*(-35), Sp^R^, Erm^R^ | AH10361 x pCF107 |
| AH10384 | *trpC2 metC3* *tgl::sp* Δ*thrC::tgl-hl4-cfp*(-35) Δ*safA* Δa*myE::safA*^M161/164A^, Sp^R^, Erm^R^, Neo^R^ | AH10357 x pCF108 |
| AH10393 | *trpC2 metC3* *tgl::sp Δthrc::tgl*^C116^_A_*-hl4-cfp*(-35), Sp^R^, Erm^R^ | AH10361 x pCF114 |
| AH10394 | *trpC2 metC3* *tgl::sp Δthrc::tgl*^H200A^*-hl4-cfp*(-35), Sp^R^, Erm^R^ | AH10361 x pCF115 |
| AH10395 | *trpC2 metC3* *tgl::sp Δthrc::tgl*^W149A^*-hl4-cfp*(-35), Sp^R^, Erm^R^ | AH10361 x pCF116 |
| AH10399 | *trpC2 metC3* *tgl::sp* Δ*thrC::tgl-hl4-cfp*(-35) Δ*safA* Δa*myE::cm*, *gerQ::neo*, Sp^R^, Erm^R^, Cm^R^, Neo^R^ | AH10357 x AH10355 |
| AH10401 | *trpC2 metC3* *tgl::sp* Δ*thrC::tgl-hl4-cfp*(-35) Δ*safA* Δa*myE::cm*, *gerQ::neo*, *yeeK::tet*, Sp^R^, Erm^R^, Cm^R^, Neo^R^, Tet^R^ | AH10399 x AH10354 |
| AH10416 | *trpC2 metC3* *tgl::sp ΔthrC::tgl*^W184A^*-hl4-cfp*(-35), Sp^R^, Erm^R^ | AH10361 x pCF121 |
| AH10417 | *trpC2 metC3* *tgl::sp ΔthrC::tgl*^R185A^*-hl4-cfp*(-35), Sp^R^, Erm^R^ | AH10361 x pCF122 |
| AH10418 | *trpC2 metC3* *tgl::sp ΔthrC::tgl*^Y171A^*-hl4-cfp*(-35), Sp^R^, Erm^R^ | AH10361 x pCF123 |
| AH10506 | *trpC2 metC3* *tgl::sp* Δ*thrC::tgl-hl4-cfp*(-35) Δ*safA*  Δa*myE::safA*^F155STOP^, Sp^R^, Erm^R^, Neo^R^ | AH10357 x pCF124 |
| AH10508 | *trpC2 metC3* *tgl::sp* Δ*thrC::tgl-hl4-cfp*(-35) *gerQ::cm*, Sp^R^, Erm^R^, Cm^R^ | AH10331 x AH2203 |
| AH10509 | *trpC2 metC3* *tgl::sp* Δ*thrC::tgl-hl4-cfp*(-35) *yeeK::cm*, Sp^R^, Erm^R^, Cm^R^ | AH10282 x pCF89 |
| AH10513 | *trpC2 metC3* *tgl::sp* Δ*safA* Δa*myE::safA*^F155STOP^, Sp^R^, Neo^R^ | AH10352 x pCF124 |
| AH10518 | *trpC2 metC3* *tgl::sp* Δ*safA* Δa*myE::safA*^F155STOP^ Δ*thrC::cm*, Sp^R^, Neo^R^, Cm^R^ | AH10513 x pCF95 |
| AH10535 | *trpC2 metC3* *tgl::sp* Δ*thrC::tgl*^Y171A^*-hl4-cfp*(-35) Δ*safA*  Δa*myE::safA*^F155STOP^, Sp^R^, Erm^R^, Neo^R^ | AH10518 x pCF123 |
| AH10573 | *trpC2 metC3* *tgl::sp* Δ*safA* Δa*myE::safA*^F155STOP^-*yfp*, Sp^R^, Neo^R^ | AH10352 x pCF175 |
| AH10574 | *trpC2 metC3* *tgl::sp* Δ*thrC::tgl-hl4-cfp*(-35) Δ*safA* Δa*myE::safA*^F155STOP^-*yfp*, Sp^R^, Erm^R^, Neo^R^ | AH10357 x pCF175 |
| AH10576 | *trpC2 metC3* *tgl::sp* Δ*thrC::cm* Δ*safA* Δa*myE::safA*^F155STOP^-*yfp*, Sp^R^, Erm^R^, Neo^R^ | AH10573 x pCF95 |
| AH10577 | *trpC2 metC3* *tgl::sp* Δ*thrC::tgl*^F69A^*-hl4-cfp*(-35) Δ*safA* Δa*myE::safA*^F155STOP^-*yfp*, Sp^R^, Erm^R^, Neo^R^ | AH10576 x pCF102 |
| AH10578 | *trpC2 metC3* *tgl::sp* Δ*thrC::tgl*^N188A^*-hl4-cfp*(-35) Δ*safA* Δa*myE::safA*^F155STOP^-*yfp*, Sp^R^, Erm^R^, Neo^R^ | AH10576 x pCF107 |
| AH10579 | *trpC2 metC3* *tgl::sp* Δ*thrC::tgl*^C116A^*-hl4-cfp*(-35) Δ*safA* Δa*myE::safA*^F155STOP^-*yfp*, Sp^R^, Erm^R^, Neo^R^ | AH10576 x pCF114 |
| AH10580 | *trpC2 metC3* *tgl::sp* Δ*thrC::tgl*^H200A^*-hl4-cfp*(-35) Δ*safA* Δa*myE::safA*^F155STOP^-*yfp*, Sp^R^, Erm^R^, Neo^R^ | AH10576 x pCF115 |
| AH10581 | *trpC2 metC3* *tgl::sp* Δ*thrC::tgl*^Y171A^*-hl4-cfp*(-35) Δ*safA* Δa*myE::safA*^F155STOP^-*yfp*, Sp^R^, Erm^R^, Neo^R^ | AH10576 x pCF123 |
| AH10605 | *trpC2 metC3* *tgl::sp* Δ*thrC::tgl-hl4-cfp*(-35) *yeeK::cm gerQ::neo*, Sp^R^, Erm^R^, Cm^R^ Neo^R^ | AH10509 x AH10355 |
| AH10606 | *trpC2 metC3 yaaHΩyaaH-gfp*, Cm^R^ | Laboratory stock |
| AH10607 | *trpC2 metC3* *tgl::sp* Δ*thrC::tgl-hl4-cfp*(-35) Δ*safA*  Δa*myE::safA*^F155STOP^ *yaaHΩyaaH-gfp*, Sp^R^, Erm^R^, Neo^R^, Cm^R^ | AH10506 x AH10606 |
| AH10608 | *trpC2 metC3* *tgl::sp* Δ*thrC::tgl*^Y171A^*-hl4-cfp*(-35) Δ*safA*  Δa*myE::safA*^F155STOP^ *yaaHΩyaaH-gfp*, Sp^R^, Erm^R^, Neo^R^, Cm^R^ | AH10535 x AH10606 |
|  |  |  |
| ***E. coli*** | | |
| *E. coli* C43 | F – *ompT hsdSB (rB- mB-) gal dcm*(DE3) | [[59](#_ENREF_59)] |
| AH4611 | *E. coli* BL21(DE3) with pLOM4, Kan^R^ | [[34](#_ENREF_34)] |
| AH4612 | *E. coli* BL21(DE3) with pCF1, Kan^R^ | “ |
| AH4613 | *E. coli* BL21(DE3) with pCF8, Kan^R^ | This study |
| AH4615 | *E. coli* BL21(DE3) with pCF9, Kan^R^ | “ |
| AH4616 | *E. coli* BL21(DE3) with pCF2, Kan^R^ | [[34](#_ENREF_34)] |
| AH10110 | *E. coli* BL21(DE3) with pCF25, Kan^R^ | This study |
| AH10111 | *E. coli* BL21(DE3) with pCF20, Kan^R^ | “ |
| AH10112 | *E. coli* BL21(DE3) with pCF21, Kan^R^ | “ |
| AH10154 | *E. coli* C43 with pCF19, Kan^R^ | “ |
| AH10211 | *E. coli* BL21(DE3) with pCF43, Kan^R^ | [[34](#_ENREF_34)] |
| AH10325 | *E. coli* BL21(DE3) with pCF68, Kan^R^ | This study |

Antibiotic resistance: Cm^R^, chloramphenicol; Erm^R^, erythromycin; Kan^R^, kanamycin; Neo^R^, neomycin; Sp^R^, spectinomycin; Tet^R^, tetracycline.
