# Supplementary material for: Temporal and spatial regulation of protein cross-linking by the pre-assembled substrates of a Bacillus subtilis spore coat transglutaminase
Source: PLoS Genet. 2019 Apr 8;15(4):e1007912. doi: 10.1371/journal.pgen.1007912 (PMC6490927; doi:10.1371/journal.pgen.1007912)
Supplement: S2 Table — (DOCX) [file pgen.1007912.s009.docx]

| **S2 Table. Plasmids used in this study.** | | |
| --- | --- | --- |
| Plasmid | Relevant genotype | Origin |
| pCF1 | *tgl*^C116A^-*his*_6_, for overproduction of Tgl^C116A^ | [[34](#_ENREF_34)] |
| pCF2 | *tgl*^H200A^-*his*_6_, for overproduction of Tgl^H200A^ | “ |
| pCF8 | *tgl*^W149A^-*his*_6_, for overproduction of Tgl^W149A^ | This study |
| pCF9 | *tgl*^N188A^-*his*_6_, for overproduction of Tgl^N188A^ | “ |
| pCF12 | *tgl*^E187A^-*his*_6_, for overproduction of Tgl^E187A^ | “ |
| pCF19 | *tgl*^F69A^-*his*_6_, for overproduction of Tgl^F69A^ | This study |
| pCF20 | *tgl*^W184A^-*his*_6_, for overproduction of Tgl^W184A^ | “ |
| pCF21 | *tgl*^R185A^-*his*_6_, for overproduction of Tgl^R185A^ | “ |
| pCF25 | *tgl*^Y171A^-*his*_6_, for overproduction of Tgl^Y171A^ | “ |
| pCF43 | *tgl*^E115^_A_-*his*_6_, for overproduction of Tgl^E115A^ | “ |
| pCF62 | *yeeK* in pUC18 | This study |
| pCF65 | *yeeK::cm*, derived from pCF62 | “ |
| pCF68 | *safA*^C30^-*his*_6_, for overproduction of C30 | “ |
| pCF75 | *safA*, for integration at *amyE* | [[34](#_ENREF_34)] |
| pCF89 | *tgl-hl4-cfp* (-35), for integration at *thrC* | “ |
| pCF95 | *thrC::cm* | “ |
| pCF100 | tgl^Δ313-594^-hl4-cfp, derived from pCF89 | This study |
| pCF102 | *tgl*^F69A^*-hl4-cfp* (-35), for integration at *thrC* | “ |
| pCF104 | *tgl*^E115A^*-hl4-cfp* (-35), for integration at *thrC* | “ |
| pCF105 | *tgl*^E187A^*-hl4-cfp* (-35), for integration at *thrC* | “ |
| pCF107 | *tgl*^N188A^*-hl4-cfp* (-35), for integration at *thrC* | “ |
| pCF108 | *safA*^M161/164A^, for integration at *amyE* | [[34](#_ENREF_34)] |
| pCF114 | *tgl*^C116A^*-hl4-cfp* (-35), for integration at *thrC* | This study |
| pCF115 | *tgl*^H200A^*-hl4-cfp* (-35), for integration at *thrC* | “ |
| pCF116 | *tgl*^W149A^*-hl4-cfp* (-35), for integration at *thrC* | “ |
| pCF121 | *tgl*^W184A^*-hl4-cfp* (-35), for integration at *thrC* | “ |
| pCF122 | *tgl*^R185A^*-hl4-cfp* (-35), for integration at *thrC* | “ |
| pCF123 | *tgl*^Y171A^*-hl4-cfp* (-35), for integration at *thrC* | “ |
| pCF124 | *safA*^F155STOP^, for integration at *amyE* | “ |
| pCF149 | *safA-yfp*, for integration at *amyE* | “ |
| pCF175 | *safA*^F155STOP^-*yfp*, for integration at *amyE* | “ |
| pCm::Nm | *cm::neo* | [[58](#_ENREF_58)] |
| pCm::Tc | *cm::tet* | “ |
| pET28a(+) | T7 expression vector, C-terminal His-tag | Novagen |
| pKL183 | *yfp* | [[60](#_ENREF_60)] |
| pLOM4 | tgl-his_6_, for overproduction of Tgl | [[30](#_ENREF_30)] |
| pMS38 | *cm* | [[48](#_ENREF_48)] |
| pUC18 | General cloning vector | New England Biolabs |
