# Supplementary material for: Temporal and spatial regulation of protein cross-linking by the pre-assembled substrates of a Bacillus subtilis spore coat transglutaminase
Source: PLoS Genet. 2019 Apr 8;15(4):e1007912. doi: 10.1371/journal.pgen.1007912 (PMC6490927; doi:10.1371/journal.pgen.1007912)
Supplement: S3 Table — (DOCX) [file pgen.1007912.s010.docx]

| **S3 Table. Oligonucleotide primers used in this study.** | |
| --- | --- |
| Name | sequence (5’ to 3’) |
| yeeK-409D | gaaatcctgatggaagcttatgttgatgtacg |
| yeeK+828R | gacaccctcgagaaaaatatcacgaatggcttc |
| yeeK+260 cat-296 D | ctcggatagcttccagttcccgatatcctgcaggcaatagttac |
| yeeK+170 cat+125 R | cccagatcacggcatacatggcgaggaattcagaaaaagaag |
| safA652D | GCAAGACCAAT**AA**CCACAACAGGAGGCTATG |
| safA684R | CATAGCCTCCTGTTGTGG**TT**ATTGGTCTTGC |
| safA-fl3 dir | ccgtccggaagaagaaaatgag***ctgggcggaggcggatcaggcggaggcggatctggcggaggcggatcagc*** |
| fl3-yfp rev | gttcttctcctttact***tgccgcagctgatccgcctccgccagatccgcctccgcctgatccgcctccgcccag*** |
| fl3-yfp D | ctgcggcaagtaaaggagaagaacttttcactg |
| yfp-TsafA R | gatttacatcgttccgaacgatcatttgtatagttcatccatgccatgtg |
| safA+477D | ggctatg**ccatgg**atggaaaatgcaaattatcc |
| safA+1176R | cgttccgaac**tcgag**ctcattttcttcttccgg |
| Tgl F69Adir | gcacaaaagcggggcgaag**gct**gccacttttttaaaaacatacggg |
| Tgl F69Arev | cccgtatgtttttaaaaaagtggc**agc**cttcgccccgcttttgtgc |
| Tgl W149dir | agaattattttatatgac**gcg**cattatgagaaattgccg |
| Tgl W149rev | tatcggcaatttctcataatg**cgc**gtcatataaaataattctgtc |
| Tgl Y171Adir | ccttggagattgtttg**gct**tttaagaatcctgaatttg |
| Tgl Y171Arev | caaattcaggattcttaaa**agc**caaacaatctccaagg |
| Tgl W184Adir | ccgcaaaaggcgcaa**gcg**agaggcgaaaatgtgatac |
| Tgl W184Arev | gtatcacattttcgcctct**cgc**ttgcgccttttgcgg |
| Tgl R185Adir | ccgcaaaaggcgcaatgg**gca**ggcgaaaatgtgatactactg |
| Tgl R185Arev | cagtagtatcacattttcgcc**tgc**ccattgcgccttttgcgg |
| Tgl N188Adir | ggcgcaatggagaggcgaa**gct**gtgatactactgggggaag |
| Tgl N188Arev | cttcccccagtagtatcac**agc**ttcgcctctccattgcgcc |
| tgl+288D | gccgccttcaaaagcgattcgggacgcccatggtcttggaatc |
| tgl+453R | ccgtttaagattccaagaccatgggcgtcccgaatcgcttttg |
| tgl+735R | gcggacgatgcggaaaagagacggaacatccag |

Underlined sequences indicate introduced restriction sites; point mutations introduced are represented in **bold**; the sequence of the *fl3* linker is shown in ***bold*** ***italic***.
